# SARS-CoV-2 Infection, Hospitalization, and Mortality in Adults With and Without Cancer

**DOI:** 10.1001/jamanetworkopen.2023.31617

**Published:** 2023-08-31

**Authors:** Seyed M. Hosseini-Moghaddam, Frances A. Shepherd, Sarah Swayze, Jeffrey C. Kwong, Kelvin K. W. Chan

**Affiliations:** 1 ICES, Toronto, Ontario, Canada; 2 Transplant-Oncology Infectious Diseases, Ajmera Transplant Program, University Health Network, Toronto, Ontario, Canada; 3 Division of Infectious Diseases, Department of Medicine, University of Toronto, Toronto, Ontario, Canada; 4 Divisions of Medical Oncology and Hematology, Department of Medicine, University of Toronto, Toronto, Ontario, Canada; 5 Princess Margaret Caner Centre, University Health Network, Toronto, Ontario, Canada; 6 Public Health Ontario, Toronto, Ontario, Canada; 7 Department of Family and Community Medicine, University of Toronto, Toronto, Ontario, Canada; 8 Dalla Lana School of Public Health, University of Toronto, Toronto, Ontario, Canada; 9 Centre for Vaccine Preventable Diseases, University of Toronto, Toronto, Ontario, Canada; 10 Odette Caner Centre, Sunnybrook Health Sciences Centre, Toronto, Ontario, Canada

## Abstract

**Importance:**

Patients with cancer are at increased risk of SARS-CoV-2–associated adverse outcomes.

**Objective:**

To determine the associations of tumor type with SARS-CoV-2 infection, hospitalization, intensive care unit (ICU) admission, and death.

**Design, Setting, and Participants:**

This retrospective, population-based cohort study included community-dwelling adults aged at least 18 years in Ontario, Canada, ICES-linked provincial health databases from January 1, 2020, to November 30, 2021. Data were analyzed from December 1, 2021, to November 1, 2022.

**Exposures:**

Cancer diagnosis.

**Main Outcomes and Measures:**

The primary outcome was SARS-CoV-2 infection, and secondary outcomes included all-cause 14-day hospitalization, 21-day ICU admission, and 28-day death following SARS-CoV-2 infection. Cox proportional hazards models were used to obtain adjusted hazard ratios (aHRs) and 95% CIs.

**Results:**

Of 11 732 108 people in the ICES-linked health databases, 279 287 had cancer (57.2% female; mean [SD] age, 65.9 [16.1] years) and 11 452 821 people did not have cancer (45.7% female; mean [SD] age, 65.9 [16.0] years). Overall, 464 574 individuals (4.1%) developed SARS-CoV-2 infection. Individuals with hematologic malignant neoplasms (33 901 individuals) were at increased risk of SARS-CoV-2 infection (aHR, 1.19; 95% CI, 1.13-1.25), 14-day hospitalization (aHR, 1.75; 95% CI, 1.57-1.96), and 28-day mortality (aHR, 2.03; 95% CI, 1.74-2.38) compared with the overall population, while individuals with solid tumors (245 386 individuals) were at lower risk of SARS-CoV-2 infection (aHR, 0.93; 95% CI, 0.91-0.95) but increased risk of 14-day hospitalization (aHR, 1.11; 95% CI, 1.05-1.18) and 28-day mortality (aHR, 1.31; 95% CI, 1.19-1.44). The 28-day mortality rate was high in hospitalized patients with hematologic malignant neoplasms (163 of 321 hospitalized patients [50.7%]) or solid tumors (486 of 1060 hospitalized patients [45.8%]). However, the risk of 21-day ICU admission in patients with hematologic malignant neoplasms (aHR, 1.14; 95% CI, 0.93-1.40) or solid tumors (aHR, 0.93; 95% CI, 0.82-1.05) was not significantly different from that among individuals without cancer. The SARS-CoV-2 infection risk decreased stepwise with increasing numbers of COVID-19 vaccine doses received (1 dose: aHR, 0.63; 95% CI, 0.62-0.63; 2 doses: aHR, 0.16; 95% CI, 0.16-0.16; 3 doses: aHR, 0.05; 95% CI, 0.04-0.06).

**Conclusions and Relevance:**

These findings highlight the importance of prioritization strategies regarding ICU access to reduce the mortality risk in increased-risk populations, such as patients with cancer.

## Key Points

**Question:**

Were there associations of tumor type with SARS-CoV-2 infection, hospitalization, intensive care unit admission, and mortality compared with individuals without cancer?

**Findings:**

In this cohort study including 11 732 108 adults in Ontario, Canada, population-level health databases, SARS-CoV-2–associated hospitalization and mortality rates were significantly higher in individuals with cancer than individuals without cancer. However, intensive care unit admission rates were not significantly different between hospitalized patients with vs without cancer.

**Meaning:**

These findings suggest that the absence of a prioritization strategy for access to critical care treatment may be a factor in significantly greater SARS-CoV-2–associated mortality in patients with cancer.

## Teaser

This cohort study assesses associations of tumor type with SARS-CoV-2 infection, hospitalization, intensive care unit admission, and death in Ontario, Canada.

## Introduction

Cancer is an important comorbidity associated with an increased risk of SARS-CoV-2 infection and COVID-19–associated adverse outcomes.^1,2^ Several individual, environmental, and social determinants increase the risk of SARS-CoV-2 infection, hospitalization, and mortality.^3,4^ Since the diversity of risk factors may introduce biased risk estimates for COVID-19–associated outcomes, the health system response to COVID-19 in patients with cancer requires characterizing individual- and population-based heterogeneity of potential contributing factors to disease outcomes.^5^ The risk associated with COVID-19 outcomes in patients with hematologic malignant neoplasms or solid tumors necessitates adjustment for different individual and social determinants.^6^ In this population-based cohort study, we examined the risk of SARS-CoV-2 infections and COVID-19–associated outcomes in the population of Ontario, Canada, and in patients with hematologic malignant neoplasms or solid tumors, considering individual and social determinants of the disease.

## Methods

### Study Design

We conducted a province-wide cohort study using administrative health care databases. All data were linked using encoded identifiers and analyzed at ICES (formerly the Institute for Clinical Evaluative Sciences), a not-for-profit research institute in Ontario, Canada. Data use and analysis were authorized under section 45 of Ontario’s Personal Health Information Protection Act and thus did not require research ethics board approval. Our results were reported according to the Strengthening the Reporting of Observational Studies in Epidemiology (STROBE) reporting guideline.

### Study Population

The study population included all adults aged at least 18 years living in Ontario, Canada, from January 1, 2020, to November 30, 2021. The maximum follow-up date was December 31, 2021. All individuals had free and universal access to SARS-CoV-2 real-time polymerase chain reaction (RT-PCR) testing and hospital and physician care. We excluded non–Ontario residents, individuals with no recorded birth date or sex, persons who died before the index date (January 1, 2020), and patients with more than 1 cancer diagnosis within the 5-year lookback period.

### Exposure and Outcomes

The exposure of interest was cancer diagnosis, identified through the Ontario Cancer Registry. The primary outcome was a positive SARS-CoV-2 RT-PCR test result.^7^ We did not consider indeterminate results as positive. The secondary outcomes included 14-day all-cause hospitalization, 21-day all-cause ICU admission, and 28-day all-cause death following a positive SARS-CoV-2 test result.

### Data Sources

We used the COVID-19 Integrated Testing Database, which contains provincial SARS-CoV-2 test results and public health surveillance data to identify patients with confirmed SARS-CoV-2 infection. We used the Ontario Registered Persons Database to determine demographic characteristics and death dates. Using the Canadian Institute for Health Information’s Discharge Abstract Database, we collected the clinical and administrative data from inpatient admissions in Ontario. We used the Ontario Health Insurance Plan data set to collect service and diagnostic information from physician-based care interactions. The definitions and data sources for comorbidities and other variables are provided in eTable 1 in Supplement 1.

### Covariates

Baseline variables included age, sex, rural vs urban residence, Public Health Unit (PHU) region, past health care utilization, long-term care (LTC) residence, and several variables extracted from the 2016 Canadian census, including income quintile, dependency (ie, concentration of people who do not have income from employment) quintile, ethnic concentration or diversity (defined as percentage of recent immigrants and percentage who self-identify as a visible minority [as defined by Statistics Canada]) quintile, housing instability quintile, Ontario Marginal Index, and deprivation quintile (inability of individuals and communities to access and attain basic material needs).^8^ We ascertained the presence of the following comorbidities: chronic obstructive pulmonary disease, diabetes, asthma, chronic kidney disease, liver disease or cirrhosis, autoimmune disorder, immunocompromised status (eg, HIV infection, solid organ transplantation), rheumatoid arthritis, inflammatory bowel disease, frailty, dementia, hypertension, ischemic heart disease, congestive heart failure, and transient ischemic attack or stroke. We also determined each person’s modified Charlson-Deyo Comorbidity Index score and their health care resource utilization band from the Johns Hopkins ACG System Version 10.

### Statistical Analysis

We compared baseline characteristics between individuals with and without underlying cancer using the χ^2^ test, *t* test, and analysis of variance. We used multivariable Cox proportional hazards models to estimate hazard ratios (HRs) and their 95% CIs for the association between cancer diagnosis and SARS-CoV-2–associated outcomes. Cancer diagnosis was modeled as a categorical variable (ie, hematologic malignant neoplasms, solid tumors, or no cancer).

Considering the fundamental role of age, sex, and vaccination status in SARS-CoV-2 infection and COVID-19 outcomes, we initially calculated partially adjusted HRs (aHRs) adjusting for age, sex, and COVID-19 vaccination status in all analyses; subsequently, a fully adjusted model calculated aHRs accounting for age, sex, comorbidities, past health care utilization, income, rurality, PHU region, Ontario Marginalization Index, and 2016 Canada census variables. All statistical analyses were 2-tailed using SAS software version 9.4 (SAS Institute). We defined *P* < .05, not adjusted for multiplicity, as the level of statistical significance. Data were analyzed from December 1, 2021, to November 1, 2022.

## Result

We identified 14 587 571 individuals living in Ontario at the start date of the cohort (January 1, 2020). After applying the exclusion criteria, our cohort included 11 732 108 individuals: 279 287 individuals with cancer, including 245 386 individuals with solid tumors (mean [SD] age, 62.5 [16.1] years; 144 480 [58.9%] female) and 33 901 individuals with hematologic malignant neoplasms (mean [SD] age, 65.9 [16.0] years; 15 509 [45.7%] female), and 11 452 821 individuals without cancer (mean [SD] age, 48.4 [18.4] years; 5 853 715 [51.1%] female) (eFigure 1 and eFigure 2 in Supplement 1). Table 1 provides the baseline characteristics of the cohort.

**Table 1.** Baseline Characteristics of the Study Cohort as of January 1, 2020

Characteristic	Individuals, No. (%)			*P* value
	Hematologic malignant neoplasms (n = 33 901)	Solid tumors (n = 245 386)	No cancer (n = 11 452 821)	
Sex				
Female	15 509 (45.7)	144 480 (58.9)	5 853 715 (51.1)	<.001
Male	18 392 (54.3)	100 906 (41.1)	5 599 106 (48.9)	
Age, mean (SD), y	65.92 (16.1)	62.54 (16.1)	48.37 (18.5)	<.001
Long-term care residence	808 (2.4)	3412 (1.4)	95 197 (0.8)	<.001
Income quintile^a^^,^^b^				
1 (lowest)	6486 (19.1)	46 580 (19.0)	2 241 578 (19.6)	<.001
2	6777 (20.0)	49 357 (20.1)	2 273 972 (19.9)	
3	6696 (19.8)	48 884 (19.9)	2 306 435 (20.1)	
4	6537 (19.3)	48 573 (19.8)	2 297 678 (20.1)	
5 (highest)	7305 (21.5)	51 377 (20.9)	2 299 854 (20.1)	
Dependency quintile^a^^,^^b^				
1 (lowest)	6443 (19.0)	48 628 (19.8)	3 042 000 (26.6)	<.001
2	5952 (17.6)	44 497 (18.1)	2 293 951 (20.0)	
3	5969 (17.6)	43 424 (17.7)	1 988 469 (17.4)	
4	6475 (19.1)	45 860 (18.7)	1 919 741 (16.8)	
5 (highest)	8788 (25.9)	60 984 (24.9)	2 104 622 (18.4)	
Deprivation quintile^a^^,^^b^				
1 (lowest)	7900 (23.3)	55 681 (22.7)	2 613 008 (22.8)	<.001
2	7018 (20.7)	51 315 (20.9)	2 384 372 (20.8)	
3	6520 (19.2)	47 277 (19.3)	2 173 085 (19.0)	
4	6192 (18.3)	45 290 (18.5)	2 084 827 (18.2)	
5 (highest)	5997 (17.7)	43 830 (17.9)	2 093 491 (18.3)	
Ethnic diversity quintile^a^^,^^b^				
1 (lowest)	6545 (19.3)	49 607 (20.2)	1 807 802 (15.8)	<.001
2	6444 (19.0)	47 815 (19.5)	1 887 018 (16.5)	
3	6330 (18.7)	46 336 (18.9)	2 031 704 (17.7)	
4	6751 (19.9)	47 704 (19.4)	2 402 600 (21.0)	
5 (highest)	7557 (22.3)	51 931 (21.2)	3 219 659 (28.1)	
Residential instability quintile^a^^,^^b^				
1 (lowest)	6156 (18.2)	44 760 (18.2)	2 430 347 (21.2)	<.001
2	6301 (18.6)	46 711 (19.0)	2 134 668 (18.6)	
3	6509 (19.2)	47 367 (19.3)	2 069 665 (18.1)	
4	6357 (18.8)	47 015 (19.2)	2 042 025 (17.8)	
5 (highest)	8304 (24.5)	57 540 (23.4)	2 672 078 (23.3)	
Rural residence	3817 (11.3)	30 224 (12.3)	1 172 493 (10.2)	<.001
Public health unit region^b^				
Central East	2382 (7.0)	19 699 (8.0)	758 427 (6.6)	<.001
Central West	6656 (19.6)	48 472 (19.8)	2 204 979 (19.3)	
Durham	1490 (4.4)	11 626 (4.7)	548 339 (4.8)	
Eastern	2353 (6.9)	18 490 (7.5)	709 894 (6.2)	
North	2365 (7.0)	16 306 (6.6)	655 339 (5.7)	
Ottawa	1922 (5.7)	15 916 (6.5)	801 610 (7.0)	
Peel	2700 (8.0)	19 182 (7.8)	1 163 994 (10.2)	
Southwest	4290 (12.7)	31 849 (13.0)	1 336 954 (11.7)	
Toronto	7047 (20.8)	44 524 (18.1)	2 303 915 (20.1)	
York	2603 (7.7)	18 808 (7.7)	940 102 (8.2)	
COVID-19 vaccination status				
Unvaccinated	6714 (19.8)	44 051 (18.0)	2 240 334 (19.6)	<.001
1 Dose	618 (1.8)	4158 (1.7)	218 618 (1.9)	
2 Doses	14 087 (41.6)	132 936 (54.2)	7 378 372 (64.4)	
3 Doses	12 482 (36.8)	64 241 (26.2)	1 615 497 (14.1)	
Past health care utilization, mean (SD)				
Hospitalizations in the past 3 y	1.04 (1.77)	0.78 (1.21)	0.17 (0.62)	<.001
Family doctor visits in the past 1 y	5.72 (6.12)	5.13 (5.46)	3.43 (4.74)	<.001
Specialist doctor visits in the past 1 y	8.51 (8.28)	7.14 (6.78)	1.76 (3.59)	<.001
Resource utilization^c^				
Nonusers, or healthy users	14 (0.04)	74 (0.03)	681 779 (5.9)	<.001
Low	40 (0.1)	653 (0.3)	803 854 (7.0)	
Moderate	2470 (7.3)	23 753 (9.7)	4 490 304 (39.2)	
High	7741 (22.8)	69 858 (28.5)	3 459 434 (30.2)	
Very high	23 636 (69.7)	151 048 (61.6)	2 017 450 (17.6)	
Frailty score, mean (SD)	2.70 (5.31)	1.30 (3.53)	0.37 (2.03)	<.001
Charlson-Deyo Comorbidity Index, mean (SD)	0.44 (0.87)	0.26 (0.66)	0.23 (0.64)	<.001
Cancer type				
Bladder	NA	13 346 (5.4)	NA	<.001
Brain	NA	1718 (0.7)	NA	
Breast	NA	45 570 (18.6)	NA	
Cervix	NA	32 367 (13.2)	NA	
Colorectal	NA	24 022 (9.8)	NA	
Esophagus	NA	1065 (0.4)	NA	
Kidney	NA	8473 (3.5)	NA	
Larynx	NA	1174 (0.5)	NA	
Liver	NA	1969 (0.8)	NA	
Lung	NA	15 705 (6.4)	NA	
Melanoma	NA	6456 (2.6)	NA	
Oropharyngeal	NA	5900 (2.4)	NA	
Ovary	NA	2979 (1.2)	NA	
Pancreas	NA	2007 (0.8)	NA	
Prostate	NA	36 200 (14.8)	NA	
Stomach	NA	2768 (1.1)	NA	
Testis	NA	1862 (0.8)	NA	
Thyroid	NA	12 893 (5.3)	NA	
Uterus	NA	10 307 (4.2)	NA	
Other solid tumors	NA	18 605 (7.6)	NA	
Leukemia	6775 (20.0)	NA	NA	
Hodgkin lymphoma	1595 (4.7)	NA	NA	
Non-Hodgkin lymphoma	11 432 (33.7)	NA	NA	
Myeloma	4218 (12.4)	NA	NA	
Other hematologic malignant neoplasms	9881 (29.1)	NA	NA	
Comorbidities				
CHF	4542 (13.4)	18 260 (7.4)	301 821 (2.6)	<.001
Active liver disease or cirrhosis	468 (1.4)	2685 (1.1)	27 584 (0.2)	<.001
Chronic kidney disease	4321 (12.7)	17 058 (7.0)	249 800 (2.2)	<.001
Hypertension	19 379 (57.2)	124 882 (50.9)	2 987 585 (26.1)	<.001
IBD	522 (1.5)	2733 (1.1)	92 393 (0.8)	<.001
Immunocompromised	1631 (4.8)	1505 (0.6)	33 762 (0.3)	<.001
Previous cardiac ischemia	3745 (11.0)	19 875 (8.1)	378 371 (3.3)	<.001
Previous TIA/stroke	1072 (3.2)	5185 (2.1)	99 739 (0.9)	<.001
Asthma	5448 (16.1)	37 982 (15.5)	1 777 402 (15.5)	.01
COPD	2925 (8.6)	19 332 (7.9)	274 685 (2.4)	<.001
Dementia	1529 (4.5)	7335 (3.0)	181 574 (1.6)	<.001
Diabetes	9395 (27.7)	59 644 (24.3)	1 507 006 (13.2)	<.001

Abbreviations: CHF, congestive heart failure; COPD, chronic obstructive pulmonary disease; IBD, inflammatory bowel disease; NA, not applicable; TIA, transient ischemic attack.

^a^ Indicates area-level measure. Income quintiles measure median neighborhood incomes. Dependency quintiles measure the area-level concentration of people who do not have income from employment. Ethnic diversity quintiles measure area-level concentrations of recent immigrants and people belonging to a visible minority group (ie, persons, other than aboriginal people, who are non-Canadian in ethnicity or non-White in race). Instability quintile measures area-level concentration of people who experience high rates of family or housing instability. Deprivation quintiles measure the inability of persons and communities to access and attain basic material needs (eg, educational attainment, quality of housing).

^b^ Missing less than 1% of data.

^c^ Resource utilization demonstrates the interaction between comorbidities and health care needs, including categories ranging from a nonuser to a high complexity of illness.

### Primary Outcome

#### Overall SARS-CoV-2 Infection

Individuals with hematologic malignant neoplasms underwent a mean (SD) of 1.81 (3.75) SARS-CoV-2 tests and those with solid tumors underwent 1.56 (3.72) tests, compared with 1.30 (3.61) tests among the noncancer population (*P* < .001). The cumulative incidence of SARS-CoV-2 infection was 7185 of 245 386 individuals (2.9%) with solid tumors, 1193 of 33 901 individuals (3.5%) with hematologic malignant neoplasms, and 456 196 of 11 452 821 individuals (4.0%) in the noncancer population (*P* < .001). Overall, 8378 individuals (1.8%) with SARS-CoV-2 infection had underlying cancer (eTable 2 in Supplement 1).

eTable 3 in Supplement 1 provides the findings of multivariable Cox proportional hazards models adjusted for cancer type (hematologic malignant neoplasms vs solid tumors), COVID-19 vaccination status (1, 2, or 3 doses vs unvaccinated), age, and sex. The results of the fully adjusted analysis are provided in Table 2. Individuals with hematologic malignant neoplasms were at a greater risk of SARS-CoV-2 infection (aHR, 1.19; 95% CI, 1.13-1.25) compared with the noncancer population, while the risk in individuals with solid tumors was lower than in the general population (aHR, 0.93; 95% CI, 0.91-0.95). Overall, there was in inverse association between the number of COVID-19 vaccine doses received and SARS-CoV-2 infection risk (1 dose: aHR, 0.63; 95% CI, 0.62-0.64; 2 doses: aHR, 0.16; 95% CI, 0.16-0.17; 3 doses: aHR, 0.05; 95% CI, 0.04-0.06). The risk of SARS-CoV-2 infection was inversely associated with income quintile (ie, greater risk in the lower income groups); we also observed a greater risk of infection in quintiles with greater deprivation, dependency, and ethnic diversity indices (Table 2). We also identified an increased hazard of a positive SARS-CoV-2 test result associated with increased household size, employment type (ie, essential workers), and geographic regions (eTable 7 in Supplement 1).

**Table 2.** Multivariable Fully Adjusted Multivariable Model of SARS-CoV-2 Infection Risk

Variable	Adjusted hazard ratio (95% CI)
Cancer status	
None	1 [Reference]
Hematologic malignant neoplasms	1.19 (1.13-1.25)
Solid tumors	0.93 (0.91-0.95)
COVID-19 vaccination status (baseline unvaccinated), No. of doses received	
0	1 [Reference]
1	0.63 (0.62-0.63)
2	0.16 (0.16-0.16)
3	0.05 (0.04-0.06)
Age, per 1-y increase	0.98 (0.98-0.99)
Male sex (vs female)	0.98 (0.98-0.99)
LTC residence	9.49 (9.32-9.66)
Income quintile^a^	
1 (lowest)	1.17 (1.14-1.18)
2	1.11 (1.10-1.13)
3	1.15 (1.14-1.16)
4	1.09 (1.07-1.10)
5 (highest)	1 [Reference]
Deprivation quintile^a^	
1 (lowest)	1 [Reference]
2	1.08 (1.07-1.09)
3	1.20 (1.19-1.22)
4	1.31 (1.29-1.32)
5 (highest)	1.49 (1.47-1.51)
Dependency quintile^a^	
1 (lowest)	1 [Reference]
2	0.95 (0.94-0.96)
3	0.94 (0.93-0.95)
4	0.92 (0.91-0.93)
5 (highest)	1.03 (1.02-1.04)
Ethnic diversity quintile^a^	
1 (lowest)	1 [Reference]
2	1.19 (1.18-1.21)
3	1.32 (1.30-1.43)
4	1.48 (1.46-1.50)
5 (highest)	1.74 (1.71-1.76)
Public health region^b^	
Central East	0.54 (0.53-0.55)
Central West	0.85 (0.84-0.86)
Durham	0.88 (0.86-0.89)
Eastern	0.41 (0.40-0.42)
North	0.43 (0.40-0.44)
Ottawa	0.67 (0.66-0.68)
Peel	1.28 (1.27-1.29)
Southwest	0.79 (0.78-0.80)
Toronto	1.17 (1.16-1.19)
York	1 [Reference]

Abbreviation: LTC, long-term care.

^a^ Income quintiles measure median neighborhood incomes. Dependency quintiles measure the area-level concentration of people who do not have income from employment. Ethnic diversity quintiles measure area-level concentrations of recent immigrants and people belonging to a visible minority group (ie, persons, other than aboriginal people, who are non-Canadian in ethnicity or non-White in race). Instability quintile measures area-level concentration of people who experience high rates of family or housing instability. Deprivation quintiles measure the inability of persons and communities to access and attain basic material needs (eg, educational attainment, quality of housing).

^b^ Missing less than 1% of data.

#### SARS-CoV-2 Infection in the Cancer Cohort

eTable 4 in Supplement 1 provides the individual and social determinants associated with positive SARS-CoV-2 test results among individuals with cancer. Ten cancer types had the highest cumulative incidence rates of SARS-CoV-2 infection, including multiple myeloma (171 of 4218 individuals [4.1%]), cervical cancer (1330 of 32 367 individuals [4.1%]), testicular cancer (77 of 1862 individuals [4.1%]), Hodgkin lymphoma (64 of 1595 individuals [4.0%]), thyroid cancer (506 of 12 893 [3.9%]), liver cancer (71 of 1969 individuals [3.6%]), leukemias (227 of 6775 individuals [3.4%]), non-Hodgkin lymphoma (374 of 11 432 individuals [3.3%]), ovarian cancer (98 of 2979 individuals [3.3%]), and kidney cancers (271 of 8473 individuals [3.2%]) (eTable 5 in Supplement 1).

In univariable analysis, patients with hematologic malignant neoplasms were more likely to develop SARS-CoV-2 infection than patients with solid tumors (HR, 1.22; 95% CI, 1.15- 1.30). We also observed a greater risk of SARS-CoV-2 infection in patients with hematologic malignant neoplasms compared with patients with solid tumors in partially adjusted multivariable analysis (aHR, 1.33; 95% CI, 1.26-1.41) (eTable 6 in Supplement 1) and in the fully adjusted model (aHR, 1.23; 95% CI, 1.16-1.30) adjusting for different individual and social determinants (eTable 7 in Supplement 1). The risk of SARS-CoV-2 infection was lower with increasing number of COVID-19 vaccine doses received (1 dose: aHR, 0.55; 95% CI, 0.51-0.60; 2 doses: aHR, 0.13; 95% CI, 0.12-0.15; 3 doses: aHR, 0.08; 95% CI, 0.04-0.15). Individuals with cancer who were LTC residents were at significantly greater risk of SARS-CoV-2 infection than individuals with cancer who were not LTC residents (aHR, 8.63; 95% CI, 7.87-9.46).

#### SARS-CoV-2 Infection Incidence Trends in the Cancer Cohort

In eight 3-month trends (eTable 8 in Supplement 1) during the study period, 1.46% to 2.74% of individuals with SARS-CoV-2 infection had underlying cancer (eFigure 3 in Supplement 1). There was no significant difference in SARS-CoV-2 cumulative incidence trends between patients with solid tumors and those with hematologic malignant neoplasms.

### Secondary Outcomes

#### Overall Hospitalization, ICU Admission, and Mortality

**Figure 1.**
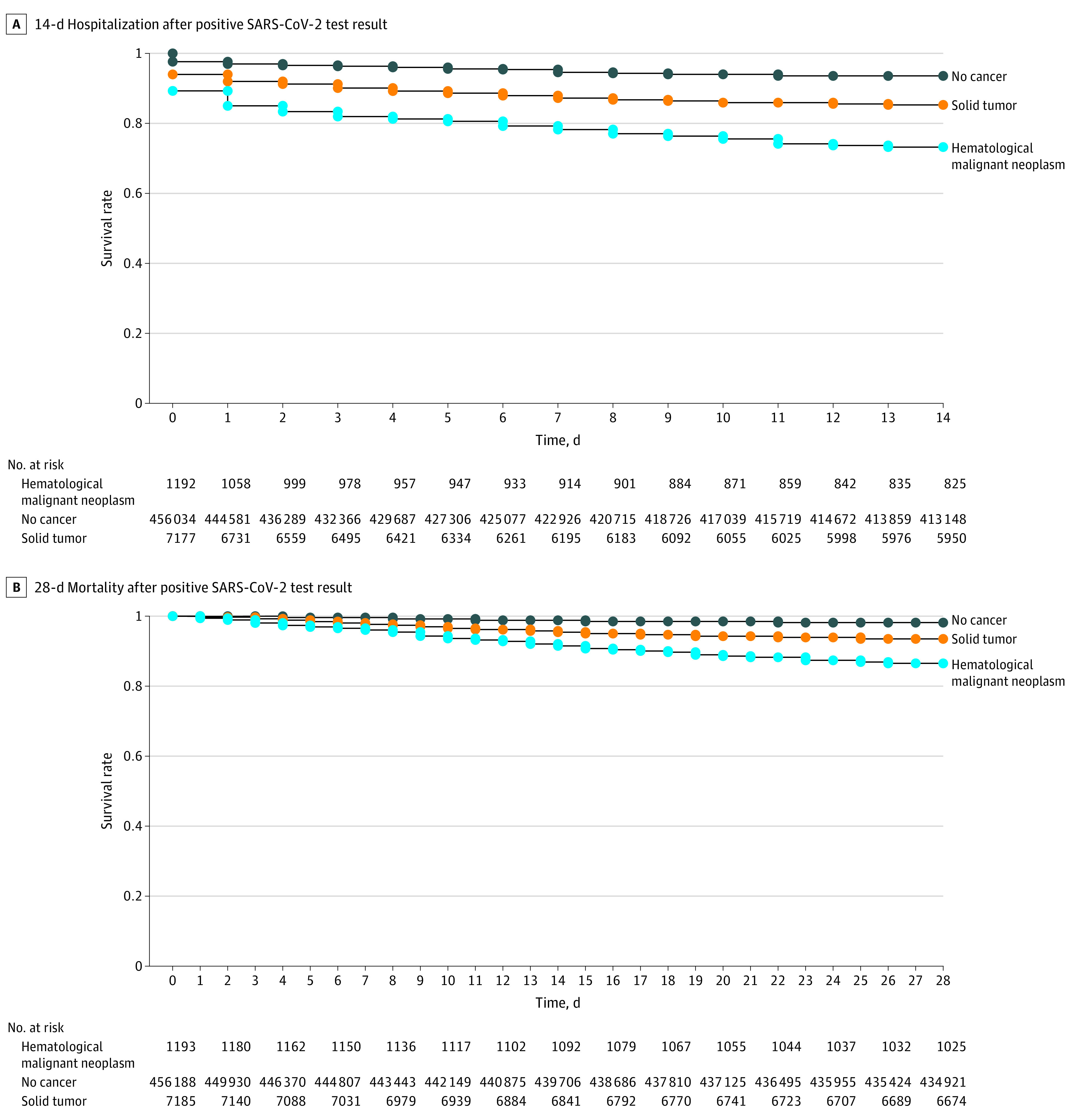
Mortality and Hospitalization Rates Following SARS-CoV-2 Infection

**Figure 2.**
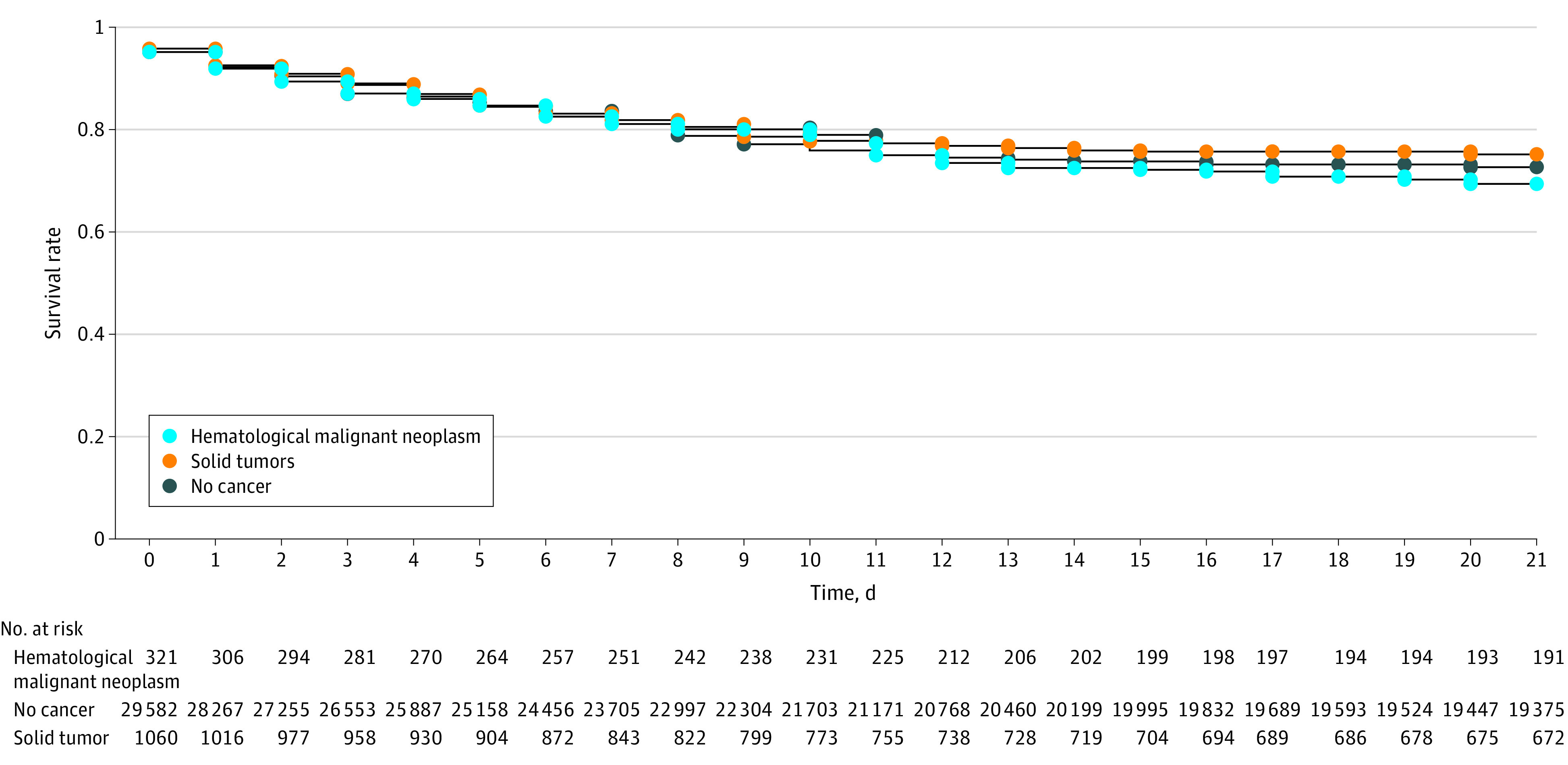
21-Day Intensive Care Unit Admission Rates After Positive SARS-CoV-2 Test Results

Compared with the noncancer population, patients with underlying cancer were at significantly greater risk of 14-day hospitalization (hematologic malignant neoplasms: HR, 4.61; 95% CI, 4.13-5.15; solid tumors: HR, 2.36; 95% CI, 2.22-2.51) (Figure 1A) and 28-day mortality (hematologic malignant neoplasms: HR, 7.67; 95% CI, 6.57-8.96; solid tumors: HR, 3.44; 95% CI, 3.14-3.77) (Figure 1B). Despite this considerable risk, the 21-day ICU admission rate was not significantly different between patients with cancer and the noncancer population (hematologic malignant neoplasms: HR, 1.12; 95% CI, 0.91-1.37; solid tumors: HR, 0.91; 95% CI, 0.81-1.03) (Figure 2). In the partially and fully adjusted models, we observed similar findings (eTable 9 and eTable 10 in Supplement 1). COVID-19 vaccination was significantly associated with decreased risk of 14-day hospitalization (1 dose: aHR, 0.68; 95% CI, 0.64-0.71; ≥2 doses: aHR, 0.42; 95% CI, 0.39-0.46), 21-day ICU admission (1 dose: aHR, 0.76; 95% CI, 0.68-0.85; ≥2 doses: aHR, 0.74; 95% CI, 0.63-0.87), and 28-day mortality (1 dose: aHR, 0.57; 95% CI, 0.51-0.63; ≥2 doses: aHR, 0.50; 95% CI, 0.45-0.57) after SARS-CoV-2 infection. We observed higher risk of hospitalization, ICU admission, and mortality with an increasing number of comorbidities measured by the modified Charlson-Deyo-Comorbidity-Index, (eTable 9 and eTable 10 in Supplement 1).

#### Hospitalization, ICU Admission, and Mortality in the Cancer Cohort

Multiple myeloma and lung cancer had the highest hospitalization and mortality rates among patients with hematologic malignant neoplasms and solid tumors (eTable 11 in Supplement 1). Male sex was also associated with COVID-19 outcomes among patients with cancer (eTable 9 and eTable 10 in Supplement 1). Among individuals with positive results in RT-PCR testing and compared with patients with solid tumors, patients with hematologic malignant neoplasms had significant higher rates of 14-day hospitalization (321 of 1193 individuals [26.9%] vs 1060 of 7185 individuals [14.8%]; *P* < .001), 21-day ICU admission (93 of 1193 individuals [7.8%] vs 256 of 7185 individuals [3.6%]; *P* < .001), and 28-day mortality (163 of 1193 individuals [13.7%] vs 486 of 7185 individuals [6.8%]; *P* < .001). However, among hospitalized patients, there were no differences among patients with hematologic malignant neoplasms and patients with solid tumors in 21-day ICU admission (93 of 321 individuals [28.9%] vs 256 of 1060 individuals [24.1%]; *P* = .08) or 28-day mortality (163 of 321 individuals [50.7%] vs 486 of 1060 individuals [45.8%]; *P* = .12).

In univariable analysis of patients with cancer and SARS-CoV-2 infection, underlying hematologic malignant neoplasms were associated with significantly greater risk of 14-day hospitalization (HR, 1.93; 95% CI, 1.70-2.19) and 28-day mortality (HR, 2.08; 95% CI, 1.74-2.49) compared with solid tumors. Hematologic malignant neoplasms were associated with greater risk of 14-day hospitalization and 28-day mortality in the partially and fully adjusted models (Table 3; eTable 12 in Supplement 1). However, the 21-day ICU admission rates were not significantly different between patients with hematologic malignant neoplasms and those with solid tumors in univariate or multivariable models. The interaction between vaccination status and cancer in the ICU outcome was not significant.

**Table 3.** Fully Adjusted Model of Risks of Hospitalization, ICU Admission, and Mortality in the Cancer Subcohort

Variable	Adjusted hazard ratio (95% CI)		
	14-d hospitalization	21-d ICU admission	28-d mortality
Hematologic malignant neoplasms (vs solid tumors)	1.57 (1.38-1.78)	1.22 (0.95-1.55)	1.65 (1.37-1.98)
COVID-19 vaccination status, No. of doses received			
0	1 [Reference]	1 [Reference]	1 [Reference]
1	0.81(0.67-0.99)	1.03 (0.70-1.51)	0.51 (0.36-0.73)
≥2	0.58 (0.44-0.76)	0.98 (0.57-1.68)	0.51 (0.33-0.76)
Age, per 1-y increase	1.05 (1.04-1.05)	0.98 (0.98-0.99)	1.07 (1.06-1.07)
Male sex (vs female)	1.36 (1.22-1.52)	1.47 (1.17-1.84)	1.61 (1.37-1.88)
LTC	0.33 (0.26-0.43)	0.59 (0.31-1.13)	1.09 (0.88-1.34)
Income quintile^a^			
1 (lowest)	0.97 (0.69-1.35)	0.63 (0.31-1.25)	1.13 (0.70-1.81)
2	0.91 (0.69-1.20)	0.62 (0.35-1.10)	0.92 (0.62-1.38)
3	1.05 (0.83-1.32)	0.98 (0.62-1.54)	1.05 (0.75-1.47)
4	1.00 (0.81-1.23)	0.86 (0.56-1.32)	0.96 (0.70-1.33)
5 (highest)	1 [Reference]	1 [Reference]	1 [Reference]
Instability quintile^a^			
1 (lowest)	1 [Reference]	1 [Reference]	1 [Reference]
2	0.99 (0.82-1.21)	1.02 (0.70-1.48)	1.08 (0.78-1.49)
3	1.06 (0.87-1.29)	0.90 (0.61-1.34)	1.00 (0.72-1.39)
4	1.05 (0.85-1.30)	0.83 (0.55-1.26)	1.25 (0.89-1.74)
5 (highest)	1.04 (0.83-1.30)	1.20 (0.78-1.85)	1.39 (0.98-1.97)
Deprivation quintile^a^			
1 (lowest)	1 [Reference]	1 [Reference]	1 [Reference]
2	1.00 (0.82-1.22)	0.88 (0.59-1.31)	0.84 (0.63-1.13)
3	1.04 (0.84-1.29)	1.12 (0.72-1.75)	1.01 (0.75-1.38)
4	1.16 (0.92-1.47)	1.18 (0.73-1.92)	1.08 (0.77-1.50)
5 (highest)	1.19 (0.92-1.55)	1.21 (0.70-2.10)	1.09 (0.75-1.58)
Dependency quintile^a^			
1 (lowest)	1 [Reference]	1 [Reference]	1 [Reference]
2	1.06 (0.90-1.25)	1.02 (0.74-1.40)	0.99 (0.75-1.30)
3	1.04 (0.87-1.24)	1.02 (0.73-1.44)	1.16 (0.88-1.52)
4	0.93 (0.78-1.12)	0.77 (0.53-1.18)	0.97 (0.73-1.28)
5 (highest)	0.82 (0.68-0.99)	0.77 (0.52-1.14)	0.84 (0.64-1.11)
Ethnic diversity quintile^a^			
1 (lowest)	1 [Reference]	1 [Reference]	1 [Reference]
2	0.89 (0.69-1.15)	1.00 (0.61-1.66)	0.93 (0.68-1.29)
3	1.10 (0.86-1.40)	1.40 (0.86-2.28)	1.01 (0.74-1.38)
4	1.09 (0.85-1.40)	1.11 (0.68-1.82)	0.85 (0.61-1.19)
5 (highest)	1.14 (0.88-1.49)	1.27 (0.75-2.14)	0.76 (0.53-1.09)
Public health region			
Central East	1.00 (0.69-1.47)	1.57 (0.78-3.16)	1.05 (0.62-1.80)
Central West	1.02 (0.81-1.28)	1.51 (0.97-2.37)	0.88 (0.62-1.24)
Durham	0.84 (0.60-1.17)	1.78 (0.96-3.33)	0.89 (0.54-1.45)
Eastern	1.06 (0.69-1.63)	3.20 (1.60-6.38)	0.79 (0.43-1.46)
North	1.02 (0.66-1.57)	1.65 (0.71-3.87)	1.11 (0.63-1.98)
Ottawa	0.81 (0.59-1.13)	1.29 (0.68-2.48)	0.85 (0.54-1.32)
Peel	0.86 (0.69-1.08)	0.97 (0.61-1.56)	0.89 (0.62-1.27)
Southwest	0.92 (0.70-1.20)	1.26 (0.72-2.18)	0.79 (0.47-1.05)
Toronto	1.04 (0.84-1.28)	1.04 (0.67-1.60)	0.86 (0.62-1.18)
York	1 [Reference]	1 [Reference]	1 [Reference]

Abbreviations: ICU, intensive care unit; LTC, long-term care facility.

^a^ Income quintiles measure median neighborhood incomes. Dependency quintiles measure the area-level concentration of people who do not have income from employment. Ethnic diversity quintiles measure area-level concentrations of recent immigrants and people belonging to a visible minority group (ie, persons, other than aboriginal people, who are non-Canadian in ethnicity or non-White in race). Instability quintile measures area-level concentration of people who experience high rates of family or housing instability. Deprivation quintiles measure the inability of persons and communities to access and attain basic material needs (eg, educational attainment, quality of housing).

## Discussion

In this population-based provincial cohort study, including more than 11 million adults living in Ontario, individuals with cancer had a significantly higher risk of SARS-CoV-2 infection and adverse COVID-19–associated outcomes compared with the noncancer population. While some cohort studies have suggested the association between cancer and COVID-19–related outcomes is due to the concomitant effect of comorbidities,^9^ the associations of solid tumors or hematologic malignant neoplasms with COVID-19–associated hospitalization and mortality in our study remained significant in analyses adjusted for sex, age, and COVID-19 vaccination and in fully adjusted models adjusted for several individual and social determinants of the disease. Among patients with solid tumors, those with lung cancer had the greatest risk of 14-day hospitalization and 28-day mortality. Lung cancer was not the most frequent cancer type among patients with cancer and SARS-CoV-2 infection, but the associations with hospitalization and mortality are likely due to underlying lung disease and a high prevalence of other smoking-related comorbidities.^10,11^

We observed higher risk of hospitalization, ICU admission, and mortality with an increasing number of comorbidities measured by the modified Charlson-Deyo-Comorbidity-Index, reflecting possible biological susceptibility to SARS-CoV-2 infection. We also identified an increased hazard of a positive SARS-CoV-2 test result associated with increased household size, employment type (ie, essential workers), geographic regions, and situational vulnerability (eg, LTC residents) consistent with findings from other cohorts, including different study populations.^3^ LTC residents were at significantly increased risk of SARS-CoV-2 infection and COVID-19–related outcomes in the cancer population. This important variable was not included in some large-scale population-based studies examining COVID-19–related outcomes in individuals with cancer.^12,13^ In the overall cohort and the cancer subcohort, we observed significant associations between SARS-CoV-2 infection and low income, deprivation, housing instability, and ethnic diversity. While the associations between these variables and COVID-19–related outcomes (ie, hospitalization and mortality) remained significant in the cohort, COVID-19–related outcomes were not significantly associated with social determinants (eg, income status, housing instability, deprivation, and ethnic diversity) in the cancer subcohort. This important finding demonstrates that although social determinants were significantly associated with modifying SARS-CoV-2 infection risk, the strong associations between underlying cancer and COVID-19–related hospitalization and death were relatively independent of social determinants. In Ontario, patients with or without cancer have relatively equitable access to health services due to a universal health care system, and this finding may not be generalizable to other jurisdictions and countries with different health care systems. Male sex was significantly associated with COVID-19–related outcomes in the cancer subcohort, likely due to a similar association in the whole cohort and a greater frequency of male sex in patients with hematologic malignant neoplasms and lung cancer.^14,15,16^

Compared with the noncancer population, individuals with hematologic malignant neoplasms had approximately 19% greater risk of SARS-CoV-2 infection, while individuals with solid tumors has approximately 7% lower risk. A lower rate of SARS-CoV-2 infection in individuals with solid tumors compared with the noncancer population is likely secondary to patient education programs and unmeasured behavioral changes (eg, wearing masks, social distancing, frequent hand washing). However, after SARS-CoV-2 infection, the 14-day hospitalization rate in individuals with hematologic malignant neoplasms (26.9%) and solid tumors (14.8%) was significantly higher than in the general population (6.5%). Following hospitalization, 28-day mortality after SARS-CoV-2 infection was significant higher in patients with hematologic malignant neoplasms (50.7%) and solid tumors (45.8%). This critical finding demonstrates that all efforts must be made to prevent SARS-CoV-2 infection and decrease the hospitalization rate in individuals with cancer and SARS-CoV-2 infection. The rapid expansion of the COVID-19 pandemic caused a considerable, continuous, and prolonged inflow of patients with severe COVID-19 and underlying cancer at substantial risk of death.^17,18^ We found that the 21-day ICU admission rates in hospitalized patients with hematologic malignant neoplasms or solid tumors were not significantly different from the rate observed in the noncancer population. This important finding is likely due to the depletion of hospital supplies and the need for more resources in the ICU setting across the province during the pandemic^19^ and highlights the need for risk-based strategies in the decision-making process for ICU transfer considering the mortality risk of patients with comorbidities, such as cancer. When critical care resources are limited, the first-come, first-served basis for accessing an ICU bed may be challenging. Patients with a low risk of death may receive critical care because they presented earlier, and others with greater mortality risk (eg, patients with cancer) may be denied access. In a risk-based approach, medical centers can use a triage protocol allocating resources based on mortality risk. The advantages and disadvantages of the risk-based strategy can be explored in any outbreaks that may occur in the future (eg, seasonal influenza outbreaks).

The risk associated with SARS-CoV-2 infection and COVID-19–related outcomes, including mortality, decreased in a stepwise manner with increasing numbers of COVID-19 vaccine doses. Despite the decrease in immunity in patients with cancer, our findings suggest that the benefits of vaccination were preserved in this immunocompromised population. Our data support the prioritization of increased-risk populations, such as patients with cancer, for vaccination to reduce COVID-19 pandemic burden.^2^

### Limitations

Our study has some limitations. We assumed that SARS-CoV-2 infection determinants did not significantly change during the cohort period, while surveillance information showed the infection risk might propagate among social networks.^20,21^ Further studies can estimate the changes in the direction and magnitude of determinants in cancer and noncancer populations throughout the pandemic. Social determinants likely cluster within PHUs, and in models adjusted for PHUs, we could not determine whether our results were affected by social determinant variations among and within PHUs. We could not determine the social determinants at the individual level, as these variables were only available at the area level.^22^ However, the directions and estimates of the associations remained relatively consistent in the partially and fully adjusted models. Our study had adequate power to measure statistical associations considering the large size of the cohort. Data on the causes of hospitalization or mortality and some relevant determinants (eg, smoking history and obesity) were unavailable in this cohort. Similarly, we did not have access to the data on patients admitted to hospice and patients’ preferences for hospitalization or ICU admission at the individual level. To address this limitation, we calculated posthospitalization mortality in addition to all-cause mortality in the cohort. Since the Ontario medication database is limited to individuals aged at least 65 years, we could not fully identify patients receiving active chemotherapies and B-cell–depleting agents.

## Conclusions

In this population-based cohort study, we estimated the SARS-CoV-2 infection risk and COVID-19–related outcomes in the noncancer population and in individuals with solid tumors and hematologic malignant neoplasms. Although the mortality risk in individuals with solid tumors or hematologic malignant neoplasms was considerably higher following COVID-19–related hospitalization, the rate of ICU admission in patients with cancer was not significantly different from the ICU admission rate measured in the noncancer population. This issue is likely due to a lack of sufficient resources during the pandemic and likely contributed to COVID-19–related mortality in hospitalized patients with cancer and SARS-CoV-2 infection. Several individual and social determinants were associated with SARS-CoV-2 infection and COVID-19–related outcomes. Our findings highlight the importance of risk-based strategies in access to critical care when the resources are limited.

## Notes

Supplement 1. **eTable 1.** Data Sources Used for Comorbidities and Medical Conditions **eTable 2.** Overall SARS-CoV-2 Infection in the Full Provincial Cohort **eTable 3.** Multivariable Analysis, Full Provincial Cohort Considering Simultaneous Effect of Cancer Type, Vaccination Status, Age and Sex **eTable 4.** Determinants of SARS-CoV-2 Positivity Among Patients With Cancer **eTable 5.** The Frequency of Cancer Types in the Cohort and the Cumulative Incidence of SARS-CoV-2 Infection **eTable 6.** Risk of SARS-CoV-2 Infection in the Cancer Subcohort, Limited Adjusted Cox Proportional Hazard Model Including Vaccination Status, Age, Sex, and Hematologic Malignancies vs Solid Tumors **eTable 7.** Risk of SARS-CoV-2 Infection in the Cancer Subcohort, Fully-Adjusted Cox Proportional Hazard Model **eTable 8.** Cumulative Incidence Trends of SARS-CoV-2 Infection in Cancer Subcohort **eTable 9.** Hospitalization, ICU Admission and Mortality Following SARS-CoV-2 Infection in the Cohort, Limited Adjusted Model (Baseline Group: Noncancer Population With SARS-CoV-2 Infection) **eTable 10.** Hospitalization, ICU Admission and Mortality Following SARS-CoV-2 Infection in the Cohort, Fully Adjusted Model (Baseline Group: Noncancer Population With SARS-CoV-2 Infection) **eTable 11.** SARS-CoV-2 Infection, Hospitalization, and Mortality in Solid Tumors and Hematologic Malignancies **eTable 12.** Risk of Hospitalization, ICU Admission, and Mortality in the Cancer Subcohort (Hematologic Malignancies vs Solid Tumor) Adjusted for Age, Sex, and COVID-19 Vaccination **eFigure 1.** Study Flow Diagram **eFigure 2.** Frequency of Cancer Types in the Cohort **eFigure 3.** Three-Month Trends of SARS-CoV-2 Infection in Cancer Subcohort

Supplement 2.
Data Sharing Statement
