# Supplementary material for: SARS-CoV-2 Infection, Hospitalization, and Mortality in Adults With and Without Cancer
Source: JAMA Netw Open. 2023 Aug 31;6(8):e2331617. doi: 10.1001/jamanetworkopen.2023.31617 (PMC10472189; doi:10.1001/jamanetworkopen.2023.31617)
Supplement: Supplement 1. — eTable 1. Data Sources Used for Comorbidities and Medical Conditions eTable 2. Overall SARS-CoV-2 Infection in the Full Provincial Cohort eTable 3. Multivariable Analysis, Full Provincial Cohort Considering Simultaneous Effect of Cancer Type, Vaccination Status, Age and Sex eTable 4. Determinants of SARS-CoV-2 Positivity Among Patients With Cancer eTable 5. The Frequency of Cancer Types in the Cohort and the Cumulative Incidence of SARS-CoV-2 Infection eTable 6. Risk of SARS-CoV-2 Infection in the Cancer Subcohort, Limited Adjusted Cox Proportional Hazard Model Including Vaccination Status, Age, Sex, and Hematologic Malignancies vs Solid Tumors eTable 7. Risk of SARS-CoV-2 Infection in the Cancer Subcohort, Fully-Adjusted Cox Proportional Hazard Model eTable 8. Cumulative Incidence Trends of SARS-CoV-2 Infection in Cancer Subcohort eTable 9. Hospitalization, ICU Admission and Mortality Following SARS-CoV-2 Infection in the Cohort, Limited Adjusted Model (Baseline Group: Noncancer Population With SARS-CoV-2 Infection) eTable 10. Hospitalization, ICU Admission and Mortality Following SARS-CoV-2 Infection in the Cohort, Fully Adjusted Model (Baseline Group: Noncancer Population With SARS-CoV-2 Infection) eTable 11. SARS-CoV-2 Infection, Hospitalization, and Mortality in Solid Tumors and Hematologic Malignancies eTable 12. Risk of Hospitalization, ICU Admission, and Mortality in the Cancer Subcohort (Hematologic Malignancies vs Solid Tumor) Adjusted for Age, Sex, and COVID-19 Vaccination eFigure 1. Study Flow Diagram eFigure 2. Frequency of Cancer Types in the Cohort eFigure 3. Three-Month Trends of SARS-CoV-2 Infection in Cancer Subcohort [file jamanetwopen-e2331617-s001.pdf]

Supplemental Online Content

Hosseini-Moghaddam SM, Shepherd FA, Swayze S, Kwong JC, Chan KW. SARS-CoV-2 infection, hospitalization, and mortality in adults with and without cancer. *JAMA Netw Open*. 2023;6(8):e2331617. doi:10.1001/jamanetworkopen.2023.31617

eTable 1. Data Sources Used for Comorbidities and Medical Conditions
eTable 2. Overall SARS-CoV-2 Infection in the Full Provincial Cohort
eTable 3. Multivariable Analysis, Full Provincial Cohort Considering Simultaneous Effect of Cancer Type, Vaccination Status, Age and Sex
eTable 4. Determinants of SARS-CoV-2 positivity among patients with cancer
eTable 5. The Frequency of Cancer Types in the Cohort and the Cumulative Incidence of SARS-CoV-2 Infection
eTable 6. Risk of SARS-CoV-2 Infection in the Cancer Subcohort, Limited Adjusted Cox Proportional Hazard Model Including Vaccination Status, Age, Sex, and Hematologic Malignancies vs Solid Tumors
eTable 7. Risk of SARS-CoV-2 Infection in the Cancer Subcohort, Fully-Adjusted Cox Proportional Hazard Model
eTable 8. Cumulative Incidence Trends of SARS-CoV-2 Infection in Cancer Subcohort
eTable 9. Hospitalization, ICU Admission and Mortality Following SARS-CoV-2 Infection in the Cohort, Limited Adjusted Model (Baseline Group: Noncancer Population With SARS-CoV-2 Infection)
eTable 10. Hospitalization, ICU Admission and Mortality Following SARS-CoV-2 Infection in the Cohort, Fully Adjusted Model (Baseline Group: Noncancer Population With SARS-CoV-2 Infection)
eTable 11. SARS-CoV-2 Infection, Hospitalization, and Mortality in Solid Tumors and Hematologic Malignancies
eTable 12. Risk of Hospitalization, ICU Admission, and Mortality in the Cancer Subcohort (Hematologic Malignancies vs Solid Tumor) Adjusted for Age, Sex, and COVID-19 Vaccination
eFigure 1. Study Flow Diagram
eFigure 2. Frequency of Cancer Types in the Cohort
eFigure 3. Three-Month Trends of SARS-CoV-2 Infection in Cancer Subcohort

This supplemental material has been provided by the authors to give readers additional information about their work.

© 2023 Hosseini-Moghaddam SM et al. *JAMA Network Open.*

eTable 1. Data sources used for comorbidities and medical conditions

Medical Condition	Definition
Asthma<br>(ICES cohort)	Asthma database was used to identify patients with asthma, based on 2 or more ambulatory care visits and/or 1 or more hospitalizations.<br><br><u>OHIP</u><br>OHIP diagnostic code: 493<br><br><u>CIHI-DAD</u><br>ICD-9 diagnostic code: 493<br>ICD-10 diagnostic codes: J45, J46
Chronic obstructive pulmonary disease (COPD)<br>(ICES specific cohort)	COPD database was used to identify patients with COPD, based on 1 or more ambulatory care visits and/or 1 or more hospitalizations. Algorithm to identify COPD patients were only validated in those ages 35 and over.(2)<br><br><u>OHIP</u><br>OHIP diagnostic codes: 491, 492, 496<br><br><u>CIHI-DAD</u><br>ICD-9 diagnostic codes: 491, 492, 496<br>ICD-10 diagnostic codes: J41, J42, J43, J44
Diabetes<br>(ICES cohort)	ODD was used to identify patients with diabetes, based on 2 OHIP diagnostic codes or 1 OHIP service code or 1 CIHI admission within 2 years.(3)<br><br><u>OHIP</u><br>OHIP diagnostic code: 250<br>OHIP service codes: Q040, K029, K030, K045, K046<br><br><u>CIHI-DAD, CIHI-SDS</u><br>ICD-9 diagnostic code: 250<br>ICD-10 diagnostic codes: E10, E11, E13, E14
Active cancer	i) Had any of the following treatments in the past 6 months:<br>- Cancer surgery – see "SurgeryIn6Month_CCI_for Active Cancer concept.xlsx" CCI codes in DAD<br>- Radiation - using %getnacrs where source = Cancer and DX10CODE1 = Z510<br>- Chemotherapy - using %getnacrs where source = Cancer and DX10CODE1 = Z511 or Z512<br>**AND** any evidence of cancer diagnosis in OCR prior to last treatment date (surgery, radiation and chemotherapy).<br><br>II) If not I) (i.e., no recent treatment for cancer), if they had a recent diagnosis – cancer diagnosis in OCR within the past year before index
Chronic kidney disease (CKD)<br>(DAD, NACRS, OHIP)	• CKD diagnosis code in DAD, NACRS, OHIP in 5 years <u>or</u><br>• At least 1 dialysis code in each of the 3 months prior to index<br>• Diagnosis and procedure codes found in concept dictionary<br><br><u>OHIP</u><br>OHIP diagnostic codes: 403, 585

© 2023 Hosseini-Moghaddam SM et al. *JAMA Network Open.*

Medical Condition	Definition
	<u>CIHI NACRS, CIHI-DAD</u><br>ICD-10 diagnostic codes: E102, E112, E132, E142, I12, I13, N08, N18, N19
	Patients who were on chronic dialysis (6) in the year before index date, identified as those with at least 2 of any of the following codes in OHIP, CIHI-DAD, or CIHI-SDS separated by at least 90 days, but less than 150 days<br><br><u>OHIP</u><br>OHIP service codes: R849, G323, G325, G326, G860, G862, G865 G863, G866, G330, G331, G332, G333, G861, G082, G083, G085, G090, G091, G092, G093, G094, G095, G096, G294, G295, G864, H540, H740<br><br><u>CIHI-DAD, CIHI-SDS</u><br>CCI procedure codes: 5195, 6698<br>CCP procedure code: 1PZ21
Advanced liver disease (Cirrhosis or Decompensated Cirrhosis)	See Lapointe-Shaw 2018 “Identifying cirrhosis…”<br><br>Any history of: look back to start of data<br><br>Cirrhosis Algorithm 9<br>2+ Physician Visit (dxcode 571) **or**<br>1+ Hospital Diagnosis CIRRHOSIS<br><u>ICD-9</u> : 456.1, 571.2, 571.5<br><u>ICD-10</u>: I85.9, I98.2, K70.3,K71.7, K74.6<br><br>Decompensated Cirrhosis Algorithm 5<br>1+ Physician Visit CIRRHOSIS (dxcode 571)<br>**and** (1+ Hospital Diagnosis or 1+ Procedure)<br><u>ICD-9</u>: 456.0, 456.2, 572.2, 572.3, 572.4, 782.4, 789.5<br><u>ICD-10</u>: I85.0, I86.4, I98.20, I98.3, K721, K729, K76.6, K76.7, R17, R18<br><u>CCI:</u> 1.NA.13.BA-FA, 1.NA.13.BA-X7, 1.NA.13.BA-BD, 1.KQ.76GP-NR, 1.OT.52.HA<br><u>CCP</u>: 1006, 6691<br><u>OHIP</u>: J057, Z591
Immunocompromised (HIV, transplant, immunosuppressive therapy)	• HIV (HIV)<br><br>• Transplant recipient (solid organ or stem cell)<br>o Solid organ transplant (CORR):<br>▪ For transplants before December 31, 2019: Use RECIPIENT_TREATMENT dataset in CORR library, and flag individuals where treatment_code=171 and treatment_date was before index date<br>▪ For transplants on/after January 1, 2020: Look for ICD-10 dx, CCI procedure, and OHIP feecodes from DAD, NACRS and OHIP for RENAL, LIVER, LUNG, HEART, PANCREAS, INTESTINE (see “All transplant types – 2021_01_19_JK.xlsx”)<br>• Use %dadsdsflags, %nacrsflags, %ohipflags<br>• Create a composite variable that includes all organs

© 2023 Hosseini-Moghaddam SM et al. *JAMA Network Open.*

Medical Condition	Definition
	o Allogenic/autologous bone marrow transplant (DAD, OHIP): Identify individuals with any of the following codes from database inception to index date:<br>▪ DAD:<br>• CCP (prcode) = 53.0<br>• CCI (incode) = 1WY19, 1LZ19HHU7, 1LZ19HHU8<br>▪ OHIP<br>• Feecode = Z426<br><br>• Sickle cell anemia: Use %getdadsds to look for any hospitalization with any diagnosis code with :<br>o ICD9: 282.6<br>o ICD10: D57.0-D57.2, D57.8<br><br>• Other immune system disorders<br>o Any hospitalization, ED visit or physician billing (DAD, NACRS, OHIP; each from database inception) with the following codes:<br>▪ DAD/NACRS:<br>• ICD-9: 273.2, 279.0, 279.1 , 279.2, 279.3, 279.8, 279.9, 289.8, (*NOTE: Only 4-digit ICD9 codes found)*<br>• ICD-10: D80, D81, D82, D83, D84, D89<br>▪ OHIP: dxcode = 279<br><br>• Immunosuppressive therapy (see DIN list: "immunomeds_antineo_25JAN2021.xlsx")<br>o >30 days (total days supplied) of oral corticosteroid in the 6 months before index<br>o Receipt of other immunocompromising drug (including antineoplastics) in the 6 months before index
Inflammatory Bowel Disease	OCCC cohort definition
Dementia/Frailty	<u>Dementia (ICES cohort):</u><br>1 hospitalization for dementia and/or 3 ambulatory visits for dementia, each separated by at least 30 days, within 2 years<br>and/or 1 prescription from ODB (9)<br><br><u>OHIP</u><br>OHIP diagnostic codes: 290, 331<br><br><u>CIHI-DAD, CIHI-SDS</u><br>ICD-9 diagnostic codes: 0461, 290.0, 290.1, 290.2, 290.3, 290.4, 294, 331.0, 331.1, 331.5<br>ICD-10 diagnostic codes: F00, F01, F02, F03, G30<br><br><u>ODB</u><br>1 prescription for a cholinesterase inhibitor

© 2023 Hosseini-Moghaddam SM et al. *JAMA Network Open.*

Medical Condition	Definition
	Frailty:<br>Hospital Frailty Risk Score - "Summarized" frailty score – Pull all DAD hospitalizations in the 5-years before index, and use %hospfrailty macro to determine "summarized" frailty score per IKN. (Gilbert 2018, Lancet)<br>NOTE: Codes used in this risk score also include frailty related conditions such as dementia and other chronic conditions that are reported separately.
Hypertension	HYPER database
History of congestive heart failure (CHF) (ICES cohort)	CHF database was used to identify patients with CHF, based on 1 CIHI NACRS, CIHI-DAD, CIHI-SDS, or OHIP claim and a second claim (from either) in 1 year. The CHF database is limited to those 40 years of age or older.(11)<br><br>OHIP<br>OHIP diagnostic code: 428<br><br>CIHI-DAD, CIHI-SDS<br>ICD-9 diagnostic code: 428<br>ICD-10 diagnostic codes: I500, I501, I509
History of TIA or Acute Ischemic Stroke	Transient Ischemic Attack:<br>CIHI-DAD and CIHI-NACRS were used to identify patients with a history of a transient ischemic attack, based on at least 1 hospitalization or ED visit with a diagnosis coded with one of the following codes:<br><br>ICD-9 diagnostic codes: 435, 3623<br>ICD-10 diagnostic codes: G450, G451, G452, G453, G458, G459, H340<br><br>Acute Ischemic Stroke (12):<br>CIHI-DAD was used to identify patients with a history of acute ischemic stroke, based on at least 1 hospitalization with a main diagnosis coded with one of the following codes:<br><br>ICD-9 diagnostic codes: 43301, 43311 43321 43331 43381 43391 434, 436<br>ICD-10 diagnostic codes: I63, I64, H34.1
History of cardiac ischemia	Cardiac ischemic disease (DAD, SDS): Any comorbidty in the past 5 years (DAD, any diagnosis field) or history of procedure in past 20 years (DAD, SDS):<br><br>Comorbidity (DAD, any dx, 5-year lookback):<br>Angina: ICD-10 diagnostic codes: I20<br>ICD-9: 413<br><br>Chronic Ischemic Heart Disease: ICD-10 diagnostic codes: I25<br>ICD-9: 4140, 4148, 4149<br><br>Myocardial infarction: ICD-10 diagnostic codes: I21, I22<br>ICD-9: 410, 411, 412

© 2023 Hosseini-Moghaddam SM et al. *JAMA Network Open.*

Medical Condition	Definition
	Procedure (DAD & SDS, 20 year lookback):<br>Coronary Artery Bypass Grafting:<br>CCI procedure codes: 1IJ76<br>CCP procedure codes: 481<br><br>Percutaneous Coronary Intervention:<br>CCI procedure codes: 1IJ50, 1IJ5, 1IJ57<br>CCP procedure codes: 4802, 4803
Influenza vaccination	OHIP: Use %getohip (nonlab) to search for any of the following feecodes from the most recent October 1 up to 14 days before index date: G590, G591, G592, Q130, Q590, Q690, Q691<br><br>ODB: Use %getodb to search for any of the following DINs from the most recent October 1 up to 14 days before index date:<br>- 2019/20: 02420643, 02420783, 02432730, 02473283<br>- 2020/21: 02420643, 02420783, 02432730, 02445646, 02494248, 09857645, 09857646<br>**NOTE: To include DINs from <65, change default parameter to age 65=F.
COVID-19 vaccination	COVaxON
Death	RPDB

© 2023 Hosseini-Moghaddam SM et al. *JAMA Network Open.*

eTable 2. Overall SARS-CoV-2 infection in the full provincial cohort

Variable	No Infection N=11,267,534	SARS-CoV-2 Infection N=464,574	P-value
Sex			0.34
F	5,775,885 (51.3%)	237,819 (51.2%)	
M	5,491,649 (48.7%)	226,755 (48.8%)	
Age, years Mean ± SD	48.89 ± 18.52	44.62 ± 18.33	<.001
LTC	85,518 (0.8%)	13,899 (3.0%)	<.001
Income quintile (baseline quintile 5)			<.001
1	2,180,735 (19.4%)	113,909 (24.5%)	
2	2,229,726 (19.8%)	100,380 (21.6%)	
3	2,263,628 (20.1%)	98,387 (21.2%)	
4	2,269,818 (20.1%)	82,970 (17.9%)	
5	2,291,077 (20.3%)	67,459 (14.5%)	
Dependency quintile (baseline quintile 1)			<.001
1	2,942,942 (26.1%)	154,129 (33.2%)	
2	2,242,800 (19.9%)	101,600 (21.9%)	
3	1,961,905 (17.4%)	75,957 (16.3%)	
4	1,906,678 (16.9%)	65,398 (14.1%)	
5	2,110,909 (18.7%)	63,485 (13.7%)	
Deprivation quintile (baseline quintile 1)			<.001
1	2,596,662 (23.0%)	79,927 (17.2%)	
2	2,358,797 (20.9%)	83,908 (18.1%)	
3	2,137,430 (19.0%)	89,452 (19.3%)	
4	2,041,147 (18.1%)	95,162 (20.5%)	
5	2,031,198 (18.0%)	112,120 (24.1%)	
Ethnic Diversity Quintile			<.001
1	1,830,395 (16.2%)	33,559 (7.2%)	
2	1,894,239 (16.8%)	47,038 (10.1%)	
3	2,019,055 (17.9%)	65,315 (14.1%)	
4	2,355,143 (20.9%)	101,912 (21.9%)	
5	3,066,402 (27.2%)	212,745 (45.8%)	
Instability quintile (baseline quintile 1)			<.001
1	2,363,266 (21.0%)	117,997 (25.4%)	
2	2,110,806 (18.7%)	76,874 (16.5%)	
3	2,051,056 (18.2%)	72,485 (15.6%)	
4	2,017,298 (17.9%)	78,099 (16.8%)	
5	2,622,808 (23.3%)	115,114 (24.8%)	
Rural	1,186,223 (10.5%)	20,311 (4.4%)	<.001

© 2023 Hosseini-Moghaddam SM et al. *JAMA Network Open.*

PHU region			<.001
Central East	765,215 (6.8%)	15,293 (3.3%)	
Central West	2,181,900 (19.4%)	78,207 (16.8%)	
Durham	539,333 (4.8%)	22,122 (4.8%)	
Eastern	720,711 (6.4%)	10,026 (2.2%)	
Missing	28,597 (0.3%)	1,278 (0.3%)	
North	664,172 (5.9%)	9,838 (2.1%)	
Ottawa	797,165 (7.1%)	22,283 (4.8%)	
Peel	1,100,720 (9.8%)	85,156 (18.3%)	
Southwest	1,332,206 (11.8%)	40,887 (8.8%)	
Toronto	2,221,850 (19.7%)	133,636 (28.8%)	
York	915,665 (8.1%)	45,848 (9.9%)	
Vaccination Information			<.001
Unvaccinated	1,869,571 (16.6%)	421,528 (90.7%)	
1st Dose	202,404 (1.8%)	20,990 (4.5%)	
2nd Dose	7,503,475 (66.6%)	21,920 (4.7%)	
3rd Dose	1,692,084 (15.0%)	136 (0.0%)	
Past healthcare utilization, Mean ± SD			
Hospitalization episodes in the past 3 years,	0.19 ± 0.64	0.23 ± 0.80	<.001
Family Doctor Visits in past 12 months	3.43 ± 4.73	4.52 ± 5.52	<.001
Specialist Doctor Visits in past 12 months	1.89 ± 3.80	1.92 ± 3.87	<.001
Resource utilization			<.001
Non-users	470,092 (4.2%)	12,846 (2.8%)	
Healthy Users	192,346 (1.7%)	6,583 (1.4%)	
Low resource utilization	776,232 (6.9%)	28,315 (6.1%)	
Moderate resource utilization	4,342,123 (38.5%)	174,404 (37.5%)	
High resource utilization	3,383,476 (30.0%)	153,557 (33.1%)	
Very high resource utilization	2,103,265 (18.7%)	88,869 (19.1%)	
Charlson Index			<.001
0	3,533,281 (31.4%)	135,063 (29.1%)	
1	428,824 (3.8%)	18,024 (3.9%)	
2	220,955 (2.0%)	11,649 (2.5%)	
Frailty score	0.39 ± 2.05	0.63 ± 3.03	<.001
Cancer			<.001
Hematologic malignancies	32,708 (0.3%)	1,193 (0.3%)	
Solid tumors	238,201 (2.1%)	7,185 (1.5%)	
No cancer	10,996,625 (97.6%)	456,196 (98.2%)	

© 2023 Hosseini-Moghaddam SM et al. *JAMA Network Open.*

Cancer type			<.001
no cancer	10,996,625 (97.6%)	456,196 (98.2%)	
Bladder	13,010 (0.1%)	336 (0.1%)	
Brain	1,671 (0.0%)	47 (0.0%)	
Breast	44,298 (0.4%)	1,272 (0.3%)	
Cervix	31,037 (0.3%)	1,330 (0.3%)	
Colorectal	23,346 (0.2%)	676 (0.1%)	
Esophagus	1,041 (0.0%)	24 (0.0%)	
Kidney	8,202 (0.1%)	271 (0.1%)	
Larynx	1,145 (0.0%)	29 (0.0%)	
Liver	1,898 (0.0%)	71 (0.0%)	
Lung	15,384 (0.1%)	321 (0.1%)	
Melanoma	6,331 (0.1%)	125 (0.0%)	
Oropharyngyeal	5,737 (0.1%)	163 (0.0%)	
Ovary	2,881 (0.0%)	98 (0.0%)	
Pancreas	1,968 (0.0%)	39 (0.0%)	
Prostate	35,200 (0.3%)	1,000 (0.2%)	
Stomach	2,699 (0.0%)	69 (0.0%)	
Testis	1,785 (0.0%)	77 (0.0%)	
Thyroid	12,387 (0.1%)	506 (0.1%)	
Uterus	10,015 (0.1%)	292 (0.1%)	
Other solid tumors	18,166 (0.2%)	439 (0.1%)	
Leukemia	6,548 (0.1%)	227 (0.0%)	
Hodgkin's lymphoma	1,531 (0.0%)	64 (0.0%)	
Non-Hodgkin's lymphoma	11,058 (0.1%)	374 (0.1%)	
Myeloma	4,047 (0.0%)	171 (0.0%)	
Other hematologic malignancies	9,524 (0.1%)	357 (0.1%)	
Comorbidities			
CHF	310,475 (2.8%)	14,148 (3.0%)	<.001
Active liver disease or cirrhosis	29,511 (0.3%)	1,226 (0.3%)	0.79
Chronic kidney disease	258,441 (2.3%)	12,738 (2.7%)	<.001
Hypertension	3,018,659 (26.8%)	113,187 (24.4%)	<.001
IBD	92,763 (0.8%)	2,885 (0.6%)	<.001
Immunocompromised: HIV or Organ transplant	35,055 (0.3%)	1,843 (0.4%)	<.001
Previous Cardiac ischemia	388,390 (3.4%)	13,601 (2.9%)	<.001
Previous TIA/stroke	101,541 (0.9%)	4,455 (1.0%)	<.001
Asthma	1,744,456 (15.5%)	76,376 (16.4%)	<.001
COPD	287,325 (2.6%)	9,617 (2.1%)	<.001
Dementia	173,697 (1.5%)	16,741 (3.6%)	<.001
Diabetes	1,504,090 (13.3%)	71,955 (15.5%)	<.001

CHF: congestive heart failure, COPD: Chronic obstructive pulmonary disease, IBD: Inflammatory bowel disease, LTC: Long-term care facility, PHU: Public Health Unit, Ontario

Area level income (socioeconomic status) quintile: quintiles 1 and 5 having the lowest and highest median neighborhood incomes. Dependency quintile: quintiles 1 and 5 representing the lowest and highest concentration of people who do not have income from employment. Ethnic diversity quintile: quintiles 1 and 5 representing the lowest and highest area-level concentrations of recent immigrants and people belonging to a visible minority group (i.e., persons, other than aboriginal people, who are non-Canadian in race or non-white in color). Instability quintile: quintiles 1 and 5 representing the lowest and highest area-level concentration of people who experience high rates of family or housing instability. Deprivation quintile: quintile 1 representing neighbourhoods with the lowest material deprivation, and quintile 5 representing neighbourhoods with the highest deprivation

© 2023 Hosseini-Moghaddam SM et al. *JAMA Network Open.*

eTable 3. Multivariable analysis, full provincial cohort considering simultaneous effect of cancer type, vaccination status, age and sex

Variable	Adjusted HR 95% CI
Hematologic malignancies vs no cancer	1.29 (1.22-1.35)
Solid tumors vs no cancer	0.95 (0.93-0.97)
Vaccination status	
1-dose vs unvaccinated	0.62 (0.61-0.63)
2-dose vs unvaccinated	0.17 (0.17-0.17)
3-dose vs unvaccinated	0.15 (0.12-0.18)
Age	0.99 (0.99-0.99)
Male vs female	0.95 (0.94-0.95)

© 2023 Hosseini-Moghaddam SM et al. *JAMA Network Open.*

eTable 4. Determinants of SARS-CoV-2 positivity among patients with cancer

Variable	No Infection N=270,909	SARS-CoV-2 Infection N=8,378	Standardized difference
Sex			
F	155,009 (57.2%)	4,980 (59.4%)	0.05
M	115,900 (42.8%)	3,398 (40.6%)	0.05
Age at index date, Mean ± SD	63.06 ± 16.10	59.61 ± 18.05	0.2
LTC	3,712 (1.4%)	508 (6.1%)	0.25
Income quintile [1]			
1	50,987 (18.8%)	2,079 (24.8%)	0.15
2	54,329 (20.1%)	1,805 (21.5%)	0.04
3	53,903 (19.9%)	1,677 (20.0%)	0.0
4	53,608 (19.8%)	1,502 (17.9%)	0.05
5	57,401 (21.2%)	1,281 (15.3%)	0.15
Dependency quintile [1]			
1	52,826 (19.5%)	2,245 (26.8%)	0.17
2	48,709 (18.0%)	1,740 (20.8%)	0.07
3	47,987 (17.7%)	1,406 (16.8%)	0.02
4	51,018 (18.8%)	1,317 (15.7%)	0.08
5	68,178 (25.2%)	1,594 (19.0%)	0.15
Deprivation quintile [1]			
1	62,096 (22.9%)	1,485 (17.7%)	0.13
2	56,820 (21.0%)	1,513 (18.1%)	0.07
3	52,231 (19.3%)	1,566 (18.7%)	0.01
4	49,754 (18.4%)	1,728 (20.6%)	0.06
5	47,817 (17.7%)	2,010 (24.0%)	0.16
Ethnic diversity quintile [1]			
1	55,375 (20.4%)	777 (9.3%)	0.32
2	53,254 (19.7%)	1,005 (12.0%)	0.21
3	51,330 (18.9%)	1,336 (15.9%)	0.08
4	52,585 (19.4%)	1,870 (22.3%)	0.07
5	56,174 (20.7%)	3,314 (39.6%)	0.42
Instability quintile [1]	49,121 (18.1%)	1,795 (21.4%)	0.08
1	51,639 (19.1%)	1,373 (16.4%)	0.07
2	52,519 (19.4%)	1,357 (16.2%)	0.08
3	51,880 (19.2%)	1,492 (17.8%)	0.03
4	63,559 (23.5%)	2,285 (27.3%)	0.09
5			

© 2023 Hosseini-Moghaddam SM et al. *JAMA Network Open.*

Rural [1]			
PHU region	33,633 (12.4%)	408 (4.9%)	0.27
Central East	21,803 (8.0%)	278 (3.3%)	0.21
Central West	53,629 (19.8%)	1,499 (17.9%)	0.05
Durham	12,724 (4.7%)	392 (4.7%)	0
Eastern	20,625 (7.6%)	218 (2.6%)	0.23
Missing	579 (0.2%)	28 (0.3%)	0.02
North	18,479 (6.8%)	192 (2.3%)	0.22
Ottawa	17,487 (6.5%)	351 (4.2%)	0.1
Peel	20,614 (7.6%)	1,268 (15.1%)	0.24
Southwest	35,328 (13.0%)	811 (9.7%)	0.11
Toronto	49,139 (18.1%)	2,432 (29.0%)	0.26
York	20,502 (7.6%)	909 (10.8%)	0.11
Vaccination			
Unvaccinated	43,516 (16.1%)	7,249 (86.5%)	1.99
1st Dose	4,163 (1.5%)	613 (7.3%)	0.28
≥ 2 Dose	223,230 (82.4%)	516 (6.2%)	1.23
Past healthcare utilization, Mean ± SD			
Hospital admissions in the past 3 years	0.80 ± 1.29	0.93 ± 1.54	0.09
Family Doctor Visits in past 12 months	5.16 ± 5.49	6.67 ± 7.10	0.24
Specialist Doctor Visits in past 12 months	7.31 ± 6.98	7.34 ± 7.45	0
Resource utilization			
Non-user, healthy-user, or low resource users	676 (0.2%)	19 (0.2%)	0.01
Moderate resource utilization	25,487 (9.4%)	736 (8.8%)	0.02
High resource utilization	75,185 (27.8%)	2,414 (28.8%)	0.02
Very high resource utilization	169,475 (62.6%)	5,209 (62.2%)	0.01
Frailty score, Mean ± SD	1.45 ± 3.76	2.19 ± 5.32	0.16
Cancer			
Hematologic malignancies	32,708 (12.1%)	1,193 (14.2%)	0.06
Solid tumors	238,201 (87.9%)	7,185 (85.8%)	0.06

© 2023 Hosseini-Moghaddam SM et al. *JAMA Network Open.*

Cancer type			
Bladder	13,010 (4.8%)	336 (4.0%)	0.04
Brain	1,671 (0.6%)	47 (0.6%)	0.01
Breast	44,298 (16.4%)	1,272 (15.2%)	0.03
Cervix	31,037 (11.5%)	1,330 (15.9%)	0.13
Colorectal	23,346 (8.6%)	676 (8.1%)	0.02
Esophagus	1,041 (0.4%)	24 (0.3%)	0.02
Kidney	8,202 (3.0%)	271 (3.2%)	0.01
Larynx	1,145 (0.4%)	29 (0.3%)	0.01
Liver	1,898 (0.7%)	71 (0.8%)	0.02
Lung	15,384 (5.7%)	321 (3.8%)	0.09
Melanoma	6,331 (2.3%)	125 (1.5%)	0.06
Oropharyngeal	5,737 (2.1%)	163 (1.9%)	0.01
Ovary	2,881 (1.1%)	98 (1.2%)	0.01
Pancreas	1,968 (0.7%)	39 (0.5%)	0.03
Prostate	35,200 (13.0%)	1,000 (11.9%)	0.03
Stomach	2,699 (1.0%)	69 (0.8%)	0.02
Testis	1,785 (0.7%)	77 (0.9%)	0.03
Thyroid	12,387 (4.6%)	506 (6.0%)	0.07
Uterus	10,015 (3.7%)	292 (3.5%)	0.01
Other solid tumors	18,166 (6.7%)	439 (5.2%)	0.06
Leukemia	6,548 (2.4%)	227 (2.7%)	0.02
Hodgkin’s lymphoma	1,531 (0.6%)	64 (0.8%)	0.02
Non-Hodgkin’s lymphoma	11,058 (4.1%)	374 (4.5%)	0.02
Myeloma	4,047 (1.5%)	171 (2.0%)	0.04
Other hematologic malignancies	9,524 (3.5%)	357 (4.3%)	0.04
Comorbidities			
CHF	21,974 (8.1%)	828 (9.9%)	0.06
Active liver disease or cirrhosis	3,029 (1.1%)	124 (1.5%)	0.03
Chronic kidney disease	20,600 (7.6%)	779 (9.3%)	0.06
Hypertension	140,042 (51.7%)	4,219 (50.4%)	0.03
IBD	3,167 (1.2%)	88 (1.1%)	0.01
Immunocompromised: HIV or Organ	3,008 (1.1%)	128 (1.5%)	0.04
transplant	22,949 (8.5%)	671 (8.0%)	0.02
Previous Cardiac ischemia	6,050 (2.2%)	207 (2.5%)	0.02
Previous TIA/stroke	42,013 (15.5%)	1,417 (16.9%)	0.04
Asthma	21,639 (8.0%)	618 (7.4%)	0.02
COPD	8,217 (3.0%)	647 (7.7%)	0.21
Dementia	66,564 (24.6%)	2,475 (29.5%)	0.11
Diabetes mellitus			

1 Missing: < 1%

IBD: Inflammatory bowel disease, COPD: Chronic obstructive pulmonary disease, LTC: Long-term care facility, PHU: Public Health Unit, Ontario

Area level income (socioeconomic status) quintile: quintiles 1 and 5 having the lowest and highest median neighborhood incomes. Dependency quintile: quintiles 1 and 5 representing the lowest and highest concentration of people who do not have income from employment. Ethnic diversity quintile: quintiles 1 and 5 representing the lowest and highest area-level concentrations of recent immigrants and people belonging to a visible minority group (i.e., persons, other than aboriginal people, who are non-Canadian in race or non-white in color). Instability quintile: quintiles 1 and 5 representing the lowest and highest area-level concentration of people who experience high rates of family or housing instability. Deprivation quintile: quintile 1 representing neighbourhoods with the lowest material deprivation, and quintile 5 representing neighbourhoods with the highest deprivation

© 2023 Hosseini-Moghaddam SM et al. *JAMA Network Open.*

eTable 5. The frequency of cancer types in the cohort and the cumulative incidence of SARS-CoV-2 infection

Cancer type	Number of patients in cohort	Number of patients with SARS-CoV-2 infection (%)
Solid tumor, cancer		
Breast	45,570	1,272 (2.8%)
Prostate	36,200	1,000 (2.8%)
Cervix	32,367	1,330 (4.1%)
Colorectal	24,022	676 (2.8%)
Lung	15,705	321 (2.0%)
Bladder	13,346	336 (2.5%)
Thyroid	12,893	506 (3.9%)
Uterus	10,307	292 (2.8%)
Kidney	8,473	271 (3.2%)
Melanoma	6,456	125 (1.9%)
Oropharyngyeal	5,900	163 (2.8%)
Ovary	2,979	98 (3.3%)
Stomach	2,768	69 (2.5%)
Pancreas	2,007	39 (1.9%)
Liver	1,969	71 (3.6%)
Testis	1,862	77 (4.1%)
Brain	1,718	47 (2.7%)
Larynx	1,174	29 (2.5%)
Esophagus	1,065	24 (2.3%)
Other solid tumors [1]	18,605	439 (2.4%)
Hematologic malignancies		
Non-Hodgkin's lymphoma	11,432	374 (3.3%)
Leukemia	6,775	227 (3.4%)
Myeloma	4,218	171 (4.1%)
Hodgkin's lymphoma	1,595	64 (4.0%)
Other hematologic malignancies [2]	9,881	357 (3.6%)
Total	279,287	8,378 (3.0%)

[1] All remaining topographic and histologic codes excluding cancers of the bladder, brain, breast, cervix, colorectal, esophagus, kidney, larynx, liver, lung, melanoma, oral cavity and pharynx, ovary, pancreas, prostate, stomach, testis, thyroid, and uterus

[2] All histologic codes excluding leukemia, Hodgkin's lymphoma, non-Hodgkin's lymphoma and myeloma

eTable 6. Risk of SARS-CoV-2 infection in the cancer subcohort, limited adjusted Cox proportional hazard model including vaccination status, age, sex, and hematologic malignancies vs solid tumors

Variable	Adjusted hazard ratio (95% CI)
Age, 1 year increase	0.99 (0.99-0.99)
Sex (male vs female)	1.01 (0.97-1.05)
Hematologic malignancies vs solid tumors	1.33 (1.26-1.41)
COVID-19 vaccination status (baseline unvaccinated	
1-dose	0.54 (0.50-0.58)
2-dose	0.14 (0.12-0.15)
3-dose	0.13 (0.07-0.23)

© 2023 Hosseini-Moghaddam SM et al. *JAMA Network Open.*

eTable 7. Risk of SARS-CoV-2 infection in the cancer subcohort, fully-adjusted Cox proportional hazard model

Variable	Adjusted hazard ratio (95% CI)
Hematologic malignancies vs solid tumors	1.23 (1.16-1.30)
COVID-19 vaccination status (baseline unvaccinated)	
1-dose	0.55 (0.51-0.60)
2-dose	0.13 (0.12-0.15)
3-dose	0.08 (0.04-0.15)
Age, 1 year increase	0.99 (0.99-0.99)
Sex (male vs female)	1.06 (1.01-1.10)
LTC residents	8.63 (7.87-9.46)
Income quintile (baseline quintile 5) [1]	
1	1.12 (0.99-1.26)
2	1.10 (1.00-1.21)
3	1.12 (1.03-1.22)
4	1.11 (1.04-1.20)
Instability quintile (baseline quintile 1) [1]	
2	0.95 (0.89-1.01)
3	0.90 (0.84-0.97)
4	0.87 (0.81-0.94)
5	0.81 (0.75-0.88)
Deprivation quintile (baseline quintile 1) [1]	
2	1.06 (0.99-1.13)
3	1.14 (1.06-1.23)
4	1.28 (1.18-1.40)
5	1.45 (1.32-1.60)
Dependency quintile (baseline quintile 1) [1]	
2	0.99 (0.93-1.05)
3	0.94 (0.88-1.00)
4	0.92 (0.86-0.98)
5	1.07 (1.00-1.15)
Ethnic diversity quintile (baseline quintile 1) [1]	
2	1.17 (1.08-1.28)
3	1.30 (1.19-1.41)
4	1.49 (1.36-1.63)
5	1.78 (1.62-1.96)
Public Health region (baseline York region)	
Central East	0.40 (0.35-0.45)
Central West	0.77 (0.71-0.84)
Durham	0.78 (0.69-0.87)
Eastern	0.34 (0.29-0.39)
North	0.34 (0.30-0.40)
Ottawa	0.56 (0.50-0.63)
Peel	1.18 (1.09-1.28)
Southwest	0.69 (0.63-0.76)
Toronto	1.03 (0.95-1.11)

© 2023 Hosseini-Moghaddam SM et al. *JAMA Network Open.*

1 Missing: < 1%
LTC: Long-term care facility, PHU: Public Health Unit, Ontario
Area level income (socioeconomic status) quintile: quintiles 1 and 5 having the lowest and highest median neighborhood incomes. Dependency quintile: quintiles 1 and 5 representing the lowest and highest concentration of people who do not have income from employment. Ethnic diversity quintile: quintiles 1 and 5 representing the lowest and highest area-level concentrations of recent immigrants and people belonging to a visible minority group (i.e., persons, other than aboriginal people, who are non-Canadian in race or non-white in color). Instability quintile: quintiles 1 and 5 representing the lowest and highest area-level concentration of people who experience high rates of family or housing instability. Deprivation quintile: quintile 1 representing neighbourhoods with the lowest material deprivation, and quintile 5 representing neighbourhoods with the highest deprivation

eTable 8. Cumulative incidence trends of SARS-CoV-2 infection in cancer subcohort

3-month trend	General Population Cohort N=4,56,240	Cancer Cohort N=8,378	Patients with hematologic malignancies N= 1,193	Patients with solid tumors N= 7,185
Jan 1st - March 31st, 2020	3,624 (0.78)	102 (0.02)	22 (0.26)	80 (0.95)
April 1st - June 30th, 2020	26,744 (5.76)	710 (0.15)	116 (1.38)	594 (7.09)
July 1st - Sept 30th, 2020	14,455 (3.11)	214 (0.05)	32 (0.38)	182 (2.17)
Oct 1st - Dec 31st, 2020	10,6276 (22.87)	1,929 (0.42)	269 (3.21)	1,660 (19.81)
Jan 1st - March 31st, 2021	122,702 (26.41)	2,386 (0.51)	322 (3.84)	2,064 (24.64)
April 1st - June 30th, 2021	133,003 (28.63)	2,187 (0.47)	289 (3.45)	1,898 (22.65)
July 1st - Sept 30th, 2021	27,980 (6.02)	451 (0.1)	74 (0.88)	377 (4.5)
Oct 1st - Nov 30th, 2021	21,456 (4.62)	399 (0.09)	69 (0.82)	330 (3.94)

eTable 9. Hospitalization, ICU admission and mortality following SARS-CoV-2 infection in the cohort, limited adjusted model (baseline group: noncancer population with SARS-CoV-2 infection)

	Adjusted HR (95% CI)		
Variable	14-day hospitalization	21-day ICU admission	28-day mortality
Hematologic malignancies vs noncancer<br>Solid tumors vs noncancer	2.07 (1.85-2.31)<br>1.31 (1.23-1.39)	1.13 (0.92-1.39)<br>0.94 (0.83-1.07)	2.08 (1.78-2.44)
COVID-19 vaccination status (baseline unvaccinated)<br>1-dose<br>≥2-dose	 0.75 (0.72-0.79)<br>0.45 (0.41-0.48)	 0.79 (0.71-0.89)<br>0.79 (0.67-0.92)	 0.53 (0.48-0.59)<br>0.49 (0.44-0.56)
Age, 1 year increase	1.05 (1.05-1.05)	0.99 (0.99-0.99)	1.10 (1.10-1.11)
Sex (male vs female)	1.47 (1.43-1.50)	1.42 (1.36-1.49)	1.91 (1.83-1.99)

eTable 10. Hospitalization, ICU admission and mortality following SARS-CoV-2 infection in the cohort, fully adjusted model (baseline group: noncancer population with SARS-CoV-2 infection)

© 2023 Hosseini-Moghaddam SM et al. *JAMA Network Open.*

	Adjusted hazard ratio (95% CI)		
Variable	14-day hospitalization	21-day ICU admission	28-day mortality
Hematologic malignancies vs noncancer	1.75(1.57-1.96)	1.14 (0.93-1.40)	2.04 (1.74-2.38)
Solid tumors vs noncancer	1.11 (1.05-1.18)	0.93 (0.82-1.05)	1.31 (1.19-1.44)
COVID-19 vaccination status (baseline: unvaccinated)			
1-dose	0.68 (0.64-0.71)	0.76 (0.68-0.85)	0.57 (0.51-0.63)
≥2-dose	0.42 (0.39-0.46)	0.74 (0.63-0.87)	0.50 (0.45-0.57)
Age, 1 year increase	1.05 (1.05-1.05)	0.99 (0.99-1.00)	1.10 (1.10-1.10)
Sex (male vs female)	1.39 (1.35-1.42)	1.41 (1.34-1.47)	1.89 (1.81-1.97)
LTC	0.29 (0.27-0.30)	0.53 (0.46-0.60)	1.24 (1.18-1.30)
Income quintile (baseline: quintile 5)			
1	1.14 (1.06-1.23)	1.05 (0.91-1.21)	1.12 (0.99-1.27)
2	1.07 (1.01-1.14)	1.03 (0.92-1.15)	1.15 (1.04-1.27)
3	1.09 (1.04-1.15)	0.97 (0.88-1.07)	1.08 (0.99-1.18)
4	1.02 (0.98-1.07)	0.98 (0.89-1.07)	1.01 (0.93-1.09)
Instability quintile (baseline: quintile 1)			
2	1.03 (0.99-1.07)	1.03 (0.95-1.11)	1.08 (0.99-1.17)
3	1.03 (0.99-1.08)	0.97 (0.89-1.05)	1.08 (0.99-1.17)
4	1.05 (1.00-1.10)	0.89 (0.82-0.97)	1.04 (0.96-1.13)
5	1.08 (1.03-1.13)	0.85 (0.77-0.92)	1.06 (0.97-1.15)
Deprivation quintile (baseline: quintile 1)			
2	1.01 (0.96-1.05)	1.07 (0.98-1.16)	1.05 (0.98-1.13)
3	1.01 (0.97-1.06)	1.19 (1.09-1.31)	1.10 (1.02-1.19)
4	1.04 (0.99-1.10)	1.22 (1.10-1.35)	1.11 (1.02-1.21)
5	1.13 (1.07-1.20)	1.25 (1.12-1.40)	1.22 (1.11-1.34)
Dependency quintile (baseline: quintile 1)			
2	1.02 (0.99-1.06)	1.04 (0.98-1.11)	1.06 (0.99-1.14)
3	1.00 (0.97-1.04)	0.95 (0.88-1.02)	1.09 (1.02-1.17)
4	0.99 (0.95-1.03)	1.00 (0.93-1.08)	1.05 (0.98-1.13)
5	0.93 (0.89-0.96)	0.91 (0.84-0.98)	1.04 (0.97-1.12)
Ethnic diversity quintile (baseline: quintile 1)			
2	1.08 (1.02-1.14)	0.93 (0.83-1.02)	1.18 (1.08-1.28)
3	1.16 (1.10-1.23)	0.86 (0.78-0.96)	1.23 (1.13-1.34)
4	1.19 (1.12-1.26)	0.82 (0.74-0.91)	1.07 (0.97-1.17)
5	1.24 (1.16-1.31)	0.88 (0.78-0.98)	1.12 (1.02-1.24)
Public Health region (baseline: York region)			
Central East	0.98 (0.90-1.06)	1.15 (0.99-1.34)	1.27 (1.11-1.45)
Central West	0.90 (0.85-0.94)	1.30 (1.18-1.43)	1.00 (0.91-1.09)
Durham	0.86 (0.81-0.93)	1.34 (1.17-1.52)	1.03 (0.91-1.17)
Eastern	0.90 (0.82-0.99)	1.65 (1.39-1.94)	1.00 (0.86-1.17)
North	1.30 (1.19-1.43)	1.28 (1.08-1.52)	1.22 (1.03-1.45)
Ottawa	0.86 (0.80-0.92)	1.31 (1.15-1.50)	1.17 (1.04-1.30)

© 2023 Hosseini-Moghaddam SM et al. *JAMA Network Open.*

Peel	0.95 (0.91-1.00)	0.96 (0.88-1.06)	0.99 (0.90-1.08)
Southwest	0.99 (0.93-1.05)	1.13 (1.01-1.27)	1.09 (0.98-1.21)
Toronto	0.98 (0.94-1.03)	1.11 (1.01-1.21)	1.05 (0.97-1.14)
Deyo-Charlson comorbidity			
2	1.40 (1.35-1.46)	1.07 (0.99-1.15)	1.41 (1.34-1.50)
≥3	1.89 (1.81-1.96)	1.07 (0.99-1.16)	1.99 (1.89-2.11)

LTC: Long-term care facility, PHU: Public Health Unit, Ontario
Area level income (socioeconomic status) quintile: quintiles 1 and 5 having the lowest and highest median neighborhood incomes. Dependency quintile: quintiles 1 and 5 representing the lowest and highest concentration of people who do not have income from employment. Ethnic diversity quintile: quintiles 1 and 5 representing the lowest and highest area-level concentrations of recent immigrants and people belonging to a visible minority group (i.e., persons, other than aboriginal people, who are non-Canadian in race or non-white in color). Instability quintile: quintiles 1 and 5 representing the lowest and highest area-level concentration of people who experience high rates of family or housing instability. Deprivation quintile: quintile 1 representing neighbourhoods with the lowest material deprivation, and quintile 5 representing neighbourhoods with the highest deprivation

eTable 11. SARS-CoV-2 infection, hospitalization, and mortality in solid tumors and hematologic malignancies

Cancer type	SARS-CoV-2 infection, N	14-day hospitalization rate, N (%)	28-day death rate, N (%)
Solid tumors			
Bladder	336	73 (21.7)	51 (15.2)
Brain	47	12 (25.5)	6 (12.8)
Breast	1,272	171 (13.4)	78 (6.1)
Cervix	1,330	44 (3.3)	6 (0.5)
Colorectal	676	138 (20.4)	64 (9.5)
Kidney	271	42 (15.5)	21 (7.7)
Liver	71	16 (22.5)	8 (11.3)
Lung	321	100 (31.2)	57 (17.8)
Melanoma	125	12 (9.6)	10 (8.0)
Prostate	1,000	205 (20.5)	81 (8.1)
Esophagus, stomach, pancreas	132	34 (25.7)	14 (10.6)
Thyroid, oropharyngeal, larynx	698	79 (11.3)	11 (1.5)
Testis, ovary, and uterus	467	59 (12.6)	21 (4.5)
Other solid tumors	439	76 (17.3)	47 (10.7)
Hematologic malignancies			
Leukemia	227	71 (31.3)	37 (16.3)
Hodgkin’s and non-Hodgkin’s lymphoma	438	82 (18.7)	41 (9.3)
Myeloma	171	60 (35.1)	31 (18.1)
Other hematologic malignancies	357	108 (30.3)	54 (15.1)

© 2023 Hosseini-Moghaddam SM et al. *JAMA Network Open.*

eTable 12. Risk of hospitalization, ICU admission, and mortality in the cancer subcohort (hematologic malignancies vs solid tumor) adjusted for age, sex, and COVDI-19 vaccination

	Adjusted HR (95% CI)		
Variable	14-day hospitalization	21-day ICU admission	28-day mortality
Hematologic malignancies vs solid tumors	1.61 (1.42-1.82)	1.18 (0.93-1.50)	1.65 (1.38-1.98)
COVID-19 vaccination status (baseline unvaccinated)			
1-dose	0.90 (0.75-1.1)	1.10 (0.76-1.60)	0.54 (0.38-0.75)
≥2-dose	0.60 (0.46-0.78)	0.97 (0.58-1.64)	0.51 (0.34-0.77)
Age, 1 year increase	1.04 (1.03-1.05)	0.98 (0.98-0.99)	1.07 (1.07-1.08)
Sex (male vs female)	1.46 (1.31-1.62)	1.40 (1.12-1.74)	1.59 (1.36-1.87)

eFigure 1. Study Flow Diagram

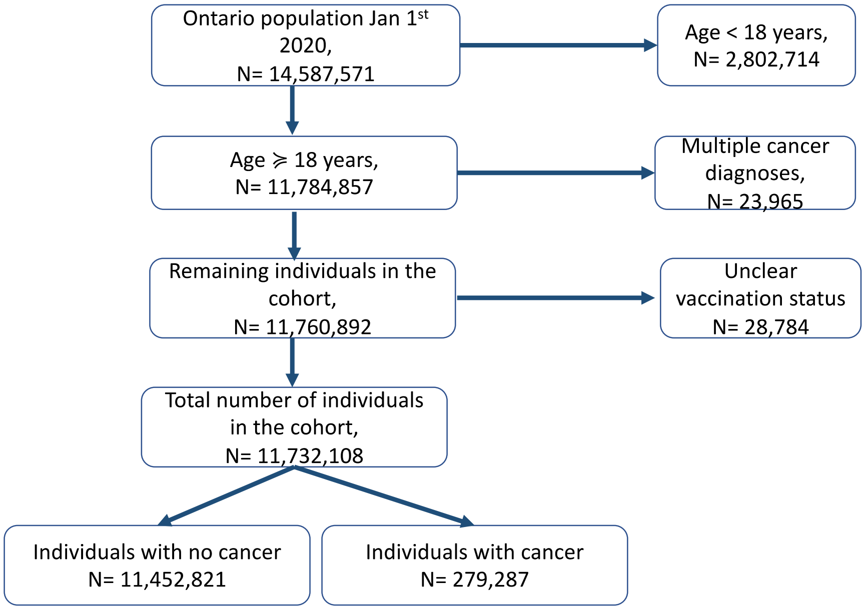

© 2023 Hosseini-Moghaddam SM et al. *JAMA Network Open.*

eFigure 2. Frequency of cancer types in the cohort

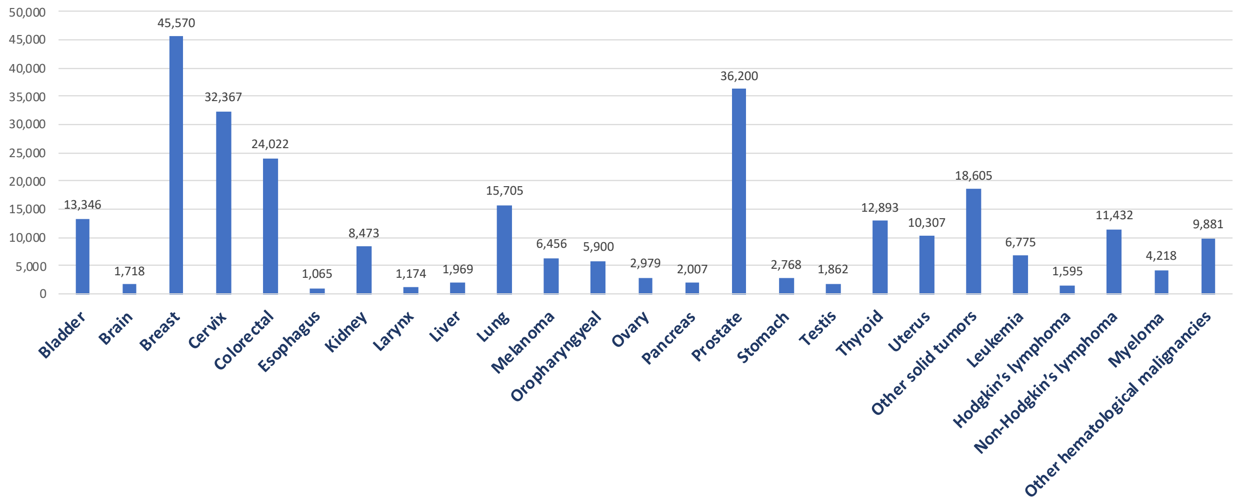

eFigure 3. three-month trends of SARS-CoV-2 infection in cancer subcohort

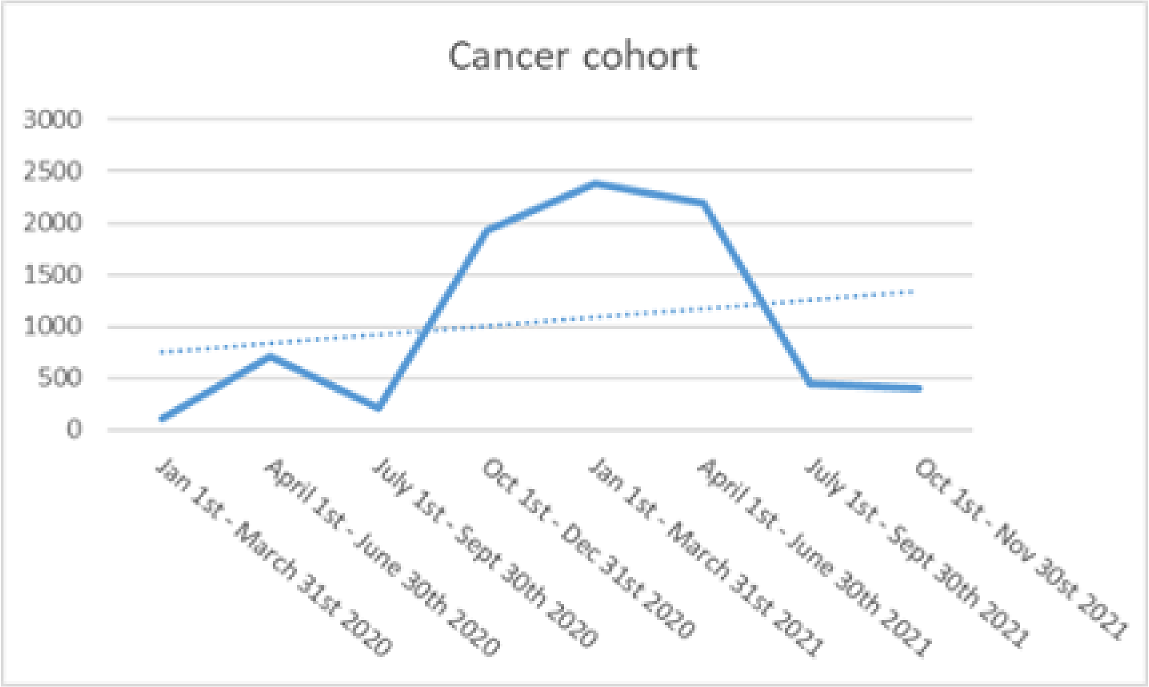

© 2023 Hosseini-Moghaddam SM et al. *JAMA Network Open.*
