# Supplementary material for: SARS-CoV-2 Infection, Hospitalization, and Mortality in Adults With and Without Cancer
Source: JAMA Netw Open. 2023 Aug 31;6(8):e2331617. doi: 10.1001/jamanetworkopen.2023.31617 (PMC10472189; doi:10.1001/jamanetworkopen.2023.31617)
Supplement: Supplement 2. — Data Sharing Statement [file jamanetwopen-e2331617-s002.pdf]

Data Sharing Statement

Hosseini-Moghaddam. SARS-CoV-2 Infection, Hospitalization, and Mortality in Adults With and Without Cancer. *JAMA Netw Open*. Published August 31, 2023.
doi:10.1001/jamanetworkopen.2023.31617

Data
Data available: No
